# Occludin-mediated barrier dysfunction and its role in cardiovascular and cerebrovascular pathogenesis

**DOI:** 10.3389/fcvm.2026.1701272

**Published:** 2026-05-08

**Authors:** Wei Xu, Tongzhang Xu, Huang Long, Qin Jiang, Weifang Liao

**Affiliations:** 1 Clinical Medical College/Affiliated Hospital of Jiujiang University, Jiujiang, Jiangxi, China; 2 Jiujiang Clinical Precision Medicine Research Center, Jiujiang, Jiangxi, China; 3 Jiangxi Provincial Clinical Research Center for Laboratory Medicine, Nanchang, Jiangxi, China; 4 Jiangxi Polytechnic University, Jiujiang, Jiangxi, China

**Keywords:** blood–brain barrier, cardiovascular, cerebrovascular pathogenesis, cerebrovascular diseases, occludin

## Abstract

Occludin (OCLN), a core transmembrane protein found in tight junctions, plays a key role in cardiovascular homeostasis by maintaining vascular endothelial barrier integrity and regulating intercellular signaling and metabolic activities. Recent evidence indicates that OCLN dysfunction is closely related to the pathological processes of various cardiovascular and cerebrovascular diseases such as ischemic stroke, atherosclerosis, and hypertensive encephalopathy. Its downregulation of expression or structural damage can directly destroy the blood–brain barrier and peripheral vascular barrier and aggravate vascular leakage, inflammatory cell infiltration, and neuronal damage; OCLN regulates the activity of signaling pathways, such as NF-κB and MAPK, through interaction with proteins such as ZO-1 and claudins, affecting inflammatory response, oxidative stress, and cell apoptosis. New evidence further reveals the non-classical function of OCLN in mediating endothelial autophagy, mitochondrial function, and metabolic reprogramming, suggesting the multidimensionality of its mechanism of action. However, the upstream and downstream regulatory networks of OCLN, tissue-specific expression differences, and their feasibility as therapeutic targets remain controversial. This systematic review examines the progress of research on the molecular mechanisms of OCLN in cardiovascular and cerebrovascular diseases, integrating its dual role in barrier damage, inflammatory cascades, and cell death, and explores potential therapeutic strategies targeting OCLN to repair barriers or regulate related signaling pathways to provide new ideas for precise intervention in cardiovascular and cerebrovascular diseases.

## 1 Introduction

Cardiovascular and cerebrovascular diseases such as stroke, atherosclerosis (AS), and hypertensive encephalopathy are among the main causes of disability and death worldwide. Their pathological mechanisms involve multiple factors, including vascular dysfunction, inflammatory response, oxidative stress, and destruction of intercellular connections (1–3). Extensive research has delved deeper into the study of vascular barrier function, with tight junction (TJ) proteins, in particular, receiving considerable attention for their role in maintaining the integrity of endothelial and epithelial barriers. Among them, OCLN, a closely linked core transmembrane protein, is the key component in the mechanical connection between cells (4) and participates in various pathophysiological processes by regulating signal transduction and cell polarity (5, 6). However, the specific mechanism of the role of OCLN in cardiovascular and cerebrovascular diseases and its clinical transformation potential have not been fully clarified, and relevant research remains controversial (7).

The stability of the blood–brain barrier (BBB) and systemic vascular endothelial barrier is highly dependent on the dynamic expression and function of OCLN. Decreased expression level or structural abnormalities of OCLN can lead to increased vascular permeability, which, in turn, causes brain edema, inflammatory cell infiltration, and neuronal damage (8, 9). This phenomenon is particularly prominent in ischemic stroke, cerebral small vascular disease, and atherosclerotic plaque instability (10). Furthermore, OCLN dysfunction may aggravate the local inflammatory response by activating NF-κB, MAPK, and other signaling pathways, forming a vicious cycle (11). Recent evidence has also revealed a non-classical role of OCLN in regulating endothelial autophagy, mitochondrial function, and metabolic reprogramming, suggesting that its role in cardiovascular and cerebrovascular diseases may far exceed that of traditionally recognized cognitive deficits (12).

Although preliminary studies have elucidated the molecular properties of OCLN and its association with disease phenotypes, the upstream and downstream regulatory networks (e.g., epigenetic modifications, post-translational modifications), tissue-specific functional differences (e.g., cerebral vs. peripheral vasculature), and feasibility of OCLN as a therapeutic target still require systematic exploration. To address this issue, this review focuses on the progress in the research on molecular mechanisms of OCLN in cardiovascular and cerebrovascular diseases, integrates its multiple roles in barrier disruption, inflammatory cascade response, and cell death, and explores novel therapeutic strategies targeting the OCLN pathway, aiming to provide a theoretical basis and direction for translational medicine research in this field.

## 2 Function and structure of OCLN

### 2.1 Molecular structure characteristics

**Figure 1:**
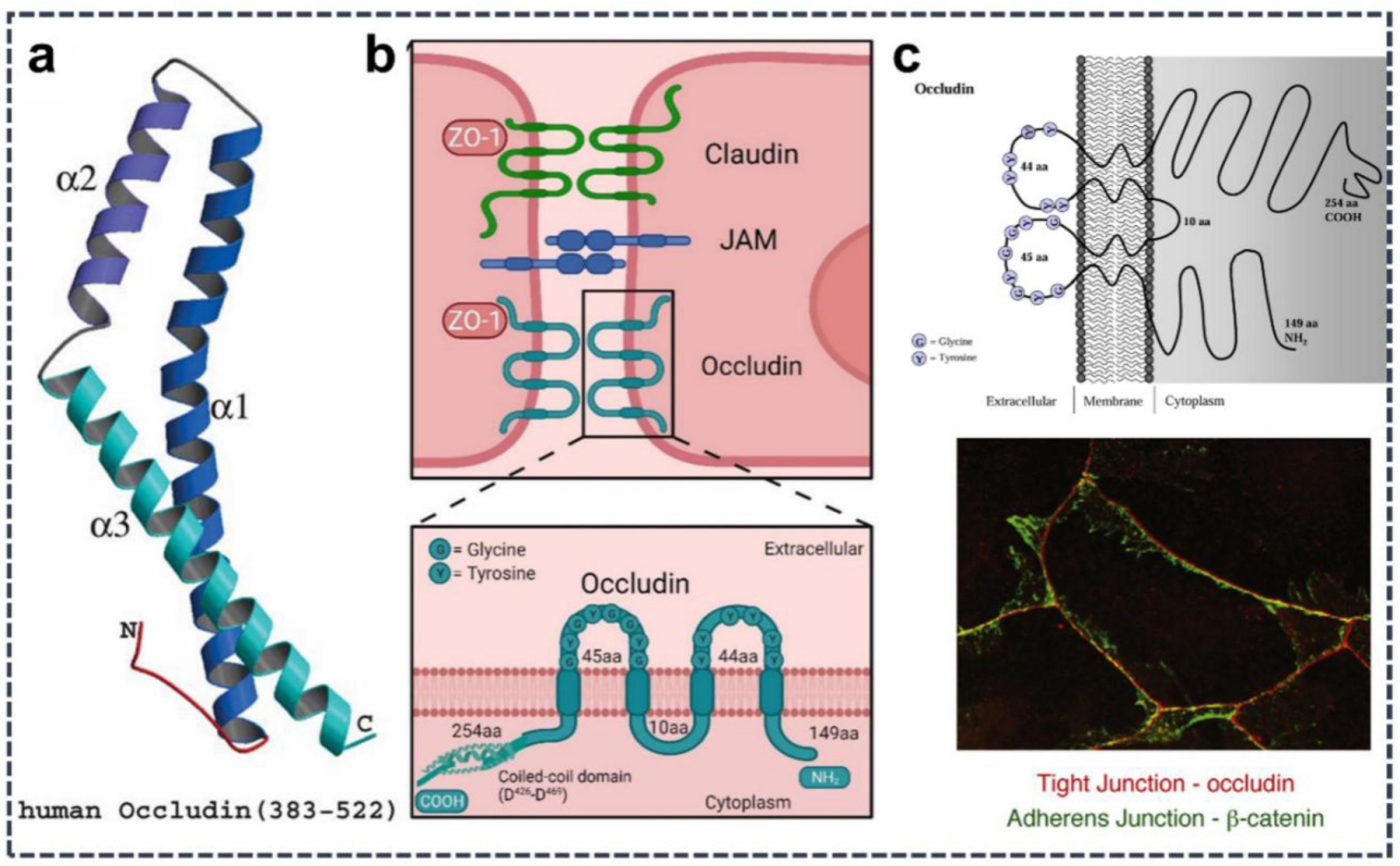
Structure and localization of occludin. **(a)** Structure of human occluding. Adapted with permission from (13). **(b)** Schematic representation of tight junctions formed by transmembrane proteins such as occludin, claudins, and junctional adhesion molecules, along with the structural domains and phosphorylation sites of occludin. Adapted with permission from (14). **(c)** Intracellular localization diagram of occludin and immunofluorescence of junctional proteins. Adapted with permission from (15, 16).

OCLN is an indispensable transmembrane protein found at the TJs of epithelial and endothelial cells. It is widely present in important organs such as the brain, liver, and kidneys. OCLN, together with proteins in the cytoplasm, such as claudins, and zonula occludens proteins (ZO-1, ZO-2, ZO-3), participates in the TJ protein composition and includes intercellular TJ proteins. OCLN is a large-molecular-weight extracellular matrix protein with a molecular weight of approximately 65 kDa (6, 13–16) (Figure 1). OCLN is the most common protein in TJs and plays a key regulatory role. Cytokines induce the stability of the paracellular osmotic barrier. OCLN has a typical transmembrane domain composed of one or more hydrophobic alpha-helical fragments so that they can be inserted into the lipid bilayer of the cell membrane. These transmembrane fragments connect the intracellular and extracellular regions of OCLN, which contribute to their stable existence and function on the cell membrane. OCLN is a protein encoded by the *OCLN* gene with four features that span the plasma membrane (17), forms two extracellular rings that simultaneously expose NH_2_ and COOH terminals to the cytoplasm, and shows a high degree of conservation over the course of biological evolution (18). Its structural characteristics include multiple specific domains such as fibronectin-like domains and epidermal growth factor-like repeats (14). The cytoplasmic tail of OCLN can bind directly to ZO-family proteins, and these protein complexes tightly attach OCLN to the actin cytoskeleton, thereby maintaining tight intercellular junctions (19). This transmembrane protein plays an important role in cellular communication and material exchange.

### 2.2 Core functional roles

The structural domain of OCLN confers its ability to interact with other extracellular matrix components and cell surface receptors, giving it unique biological functions and regulatory roles in the intra- and extracellular environments. Its main functions include intercellular connectivity, maintenance of barrier integrity, selective substance transport, and signal transduction. It can promote the tight connection between cells, stabilize intercell interactions, and maintain the integrity of the barrier; in addition, it is able to selectively transport substances to ensure the accuracy and efficiency of intracellular and extracellular substance exchange. Furthermore, OCLN plays an important role in intercellular signaling, which can rapidly and accurately transmit intercellular information and regulate the physiological activities of cells. Therefore, OCLN plays an indispensable role in maintaining the normal physiological function of cells. It is also involved in physiological and pathological processes such as wound healing, tissue regeneration, and tumor growth and metastasis (20, 21). OCLN can control cellular permeability by regulating intercellular junctions and function as a barrier, also preventing cells from spreading to the apical and basolateral membranes, thus participating in the formation of cell polarity. OCLN is mainly involved in the TJs between cells, maintaining tissue integrity and stability. In cellular communication and signaling, it regulates the exchange of substances and information between cells to ensure maintenance of normal intercellular function. In addition, OCLN has a protective effect, preventing the invasion of harmful substances and pathogens and maintaining the health of the organism (22, 23). Under pathological conditions, aberrant expression or dysfunction of OCLN may lead to the development of a range of disorders, including inflammation and tumors. Therefore, an in-depth study and understanding of the functional roles of OCLN is of great significance for maintaining human health and disease prevention (24).

### 2.3 Regulation of the expression and function of OCLN

The mechanisms regulating of OCLN expression and function involve multiple levels. Post-translational modification is an important way of regulating OCLN function, which is regulated by various post-translational modifications, among which phosphorylation (Table 1) is the most common. For instance, (Table 1) protein kinase C (PKC) and mitogen-activated protein kinase (MAPK) can phosphorylate specific serine/threonine residues of OCLN, such as Ser490 and Thr404, thereby affecting its binding ability with TJ scaffold proteins such as ZO-1 and further regulating epithelial barrier function. In addition, acetylation modification, that is (Table 1), the modification mediated by histone deacetylase (HDAC), also affects the stability of OCLN (Table 1) and its localization on the cell membrane (25). Furthermore, the Wnt/β-catenin signaling pathway plays a crucial role in the expression regulation of OCLN. Studies have shown that the nuclear translocation of β-catenin can inhibit the transcription of OCLN, thereby weakening the integrity of TJs. This mechanism has been confirmed in intestinal epithelial barrier dysfunction and tumor metastasis. In addition (Table 1), the TGF-β/Smad pathway can downregulate the expression of OCLN through a Smad4-dependent manner, promoting epithelial–mesenchymal transition (EMT) (26). Furthermore, environmental factors such as inflammation and hypoxia can also affect the expression and function of OCLN. Proinflammatory cytokines such as TNF-α and IL-6 can upregulate OCLN expression by activating the (Table 1) NF-κB signaling pathway, thereby enhancing barrier function to cope with inflammatory stimuli. However, chronic inflammation may lead to the degradation of OCLN; matrix metalloproteinase (Table 1) (MMP-9) can cleave OCLN and disrupt the BBB (Table 1). The hypoxic environment regulates OCLN expression through a HIF-1α-dependent mechanism. For example, in the ischemic stroke model, the activation of HIF-1α can reduce OCLN expression and increase the permeability of the BBB (27). Under pathological conditions, the abnormal regulation of OCLN is closely associated with various diseases. For instance, in cardiovascular diseases, the decreased expression of OCLN can lead to increased vascular endothelial permeability and exacerbate atherosclerosis; while in neurodegenerative diseases, such as Alzheimer's disease (AD), the disruption of the BBB is closely related to the degradation of OCLN (28). Therefore, an in-depth study of the regulatory mechanism of OCLN not only helps in understanding its physiological function but also provides potential targets for the treatment of related diseases.

**Table 1:** Post-translational modification and signaling pathway regulation of OCLN.

Kinases/signaling pathways	Target residue/regulatory mechanism	Functional results
PKC (231)	Ser490, Thr404 phosphorylation	Affects the binding capacity of OCLN and ZO-1, thereby regulating epithelial barrier function
MAPK (232)	Phosphorylation	Influences the phosphorylation status of OCLN, regulating barrier function and inflammatory responses
Wnt/β-catenin (234)	Inhibition of OCLN transcription	Weakening tight junction integrity promotes epithelial EMT; upregulating OCLN expression in ischemic stroke reduces BBB leakage
TGF-β/Smad (235)	Smad4-dependent downregulation of OCLN expression	Promotes EMT and participates in vascular fibrosis
NF-κB (237)	Inflammatory factors (TNF-α, IL-6) activation	Upregulating OCLN expression enhances BBB function; chronic inflammation promotes OCLN degradation
JAK-STAT3 (238)	IL-6 upregulates OCLN via STAT3	OCLN expression increases during the acute phase; it shifts to inhibition during the chronic inflammatory phase
Nrf2/ARE (239)	Low concentrations of ROS maintain basal OCLN expression through this pathway	Maintains BBB integrity and resists oxidative stress
HIF-1α (236)	Hypoxia-dependent regulation of OCLN expression	Acute hypoxia upregulates OCLN, while sustained hypoxia downregulates its expression, affecting BBB integrity
MMP-9 (240)	Cleavage of the OCLN protein	Degradation of OCLN disrupts the integrity of the BBB
HDAC (243)	Histone deacetylation affects OCLN stability	Influences the localization and expression of OCLN on the cell membrane
RhoA/ROCK (233)	Phosphorylated ZO-1 affects OCLN binding	Increases BBB permeability and promotes endothelial barrier dysfunction
PI3K/Akt (242)	Aβ inhibits this pathway, downregulating OCLN	Promotes increased BBB permeability and participates in AD pathology
FGFR1/PKCη (244)	Phosphorylation of OCLN at tyrosine 408	Enhances GLUT1 polarized localization to promote glucose transport
SIRT3/FoxO3a (246)	Inhibiting OCLN ubiquitination and degradation	Maintains OCLN stability and reduces endothelial cell apoptosis
AMPK (241)	Inhibition of MLCK-mediated OCLN endocytosis	Preserves the integrity of the endothelial barrier and mitigates inflammatory damage
mTORC2/RhoA (245)	Inhibition of OCLN endocytosis	Enhances vascular regeneration and endothelial function

BBB, blood–brain barrier; EMT, epithelial–mesenchymal transition.

## 3 OCLN in endothelial barrier function

### 3.1 Positioning in the cerebrovascular system

#### 3.1.1 High expression and functional correlation in the BBB

**Figure 2:**
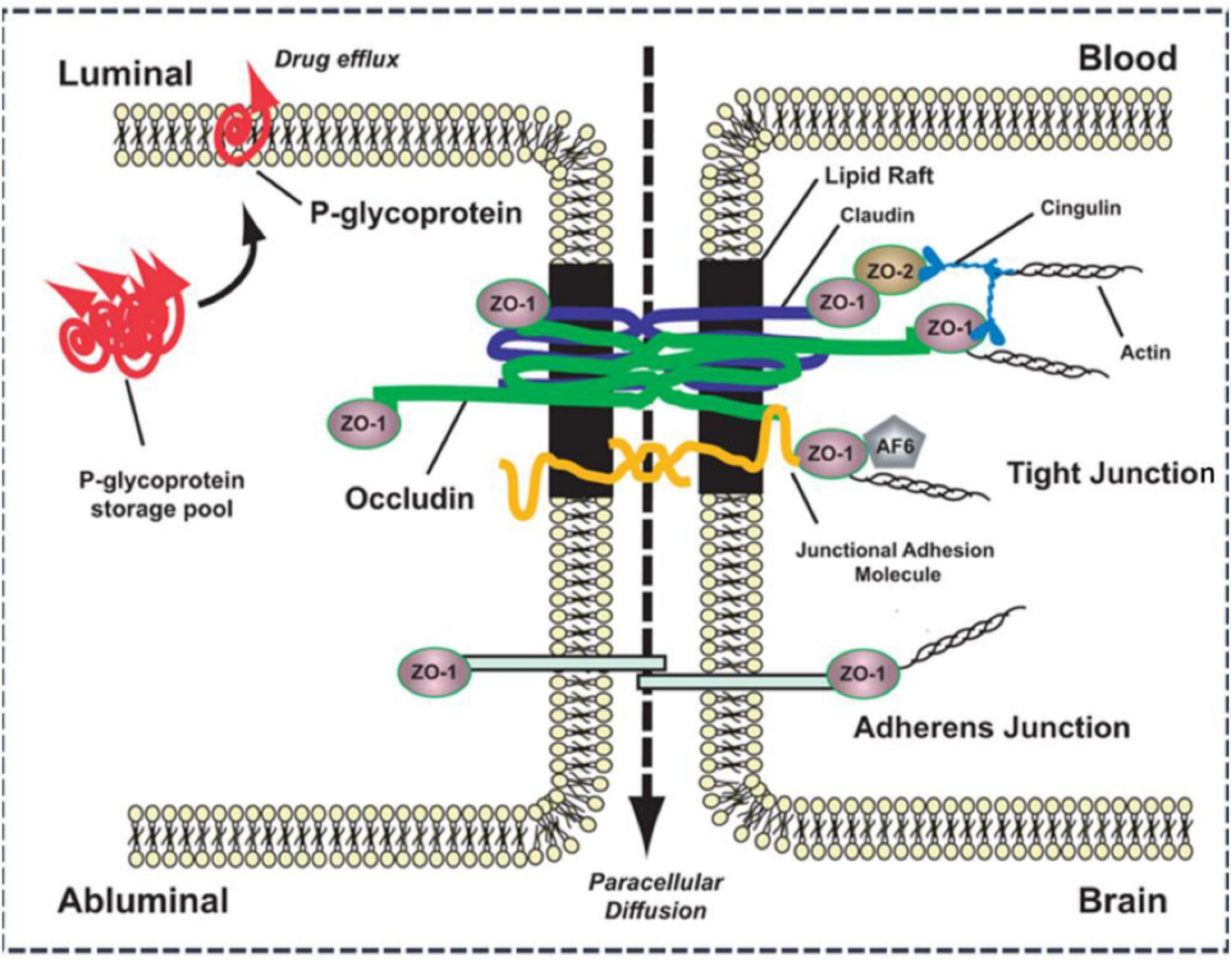
Fundamental molecular structure of tight junctions in the blood–brain barrier. Adapted with permission from (30).

The physical barrier formed by brain microvascular endothelial cells (BMECs) is the core structural basis for the selective permeability of the BBB. Among them, OCLN, one of the earliest identified transmembrane TJ proteins, shows a highly specific expression pattern in BMECs. Immunofluorescence and protein blotting analyses indicate that OCLN is significantly upregulated during the maturation stage of the BBB. Its expression level is positively correlated with barrier function (29). OCLN plays a crucial role in maintaining the integrity and permeability of the BBB by regulating the TJs between cells (30) (Figure 2). Highly expressed OCLN enhances the TJs between endothelial cells, thereby forming an effective barrier that prevents harmful substances and pathogens from entering the brain. At the same time, it regulates the transportation of nutrients to the brain, ensuring an adequate supply of nutrients to the central nervous system (31). Under physiological conditions, a high expression of OCLN can maintain the permeability of the BBB within the normal range. However, when the expression of OCLN is damaged or downregulated, the permeability of the BBB changes, which may lead to the invasion of harmful substances and pathogens into the brain and trigger a series of neurological diseases. Therefore, a high expression of OCLN in the endothelial cells of the BBB is essential for maintaining the stability and permeability of the BBB. By interacting with tight junction proteins such as claudins and cadherins, OCLN forms a “fence” effect that precisely controls the flow of substances across the BBB (32).

At the transcriptional level, gene expression of OCLN is regulated by a variety of transcription factors. These transcription factors are activated or repressed under specific conditions, thus regulating the synthesis of OCLN. In addition, OCLN undergoes a series of post-translational modification processes such as phosphorylation and glycosylation, which can alter its function or localization. Together, these mechanisms maintain the stability and permeability of the BBB, ensuring normal function of the central nervous system (33). In summary, the high expression of OCLN in BBB endothelial cells is closely related to the function of the BBB, which maintains the stability and permeability of the BBB by regulating the TJs between cells, thus protecting the central nervous system from harmful substances. The expression level of OCLN also directly affects BBB permeability, which plays an important role in maintaining the stability and health of the internal human environment.

#### 3.1.2 Differences in distribution and physiological significance of different brain regions

The distributional differences and functional specificities of different brain regions are the basis for the diversity and complexity of brain functions. These differences are reflected not only in the structure and location but also in their respective functions, which together maintain the normal physiological activities. The distributional differences and functional specificity of different brain regions are important components of the nervous system. For example, the hippocampus and cortex perform specific physiological functions and are differentially distributed in the brain, accordingly (34, 35). The hippocampus, located primarily in the medial temporal lobe, is closely associated with memory formation and storage. It has a dense distribution of neurons, forming a complex network structure that helps process and store spatial information, short-term memory, and emotional responses. In contrast, the cortex covers the surface of the brain and has a wide distribution and diverse functions. The cortex is involved in many higher neural activities such as sensory, motor, cognitive, and emotional activities and is an important area for processing complex information and performing high-level thinking activities (36). The distribution differences in these brain regions are reflected not only in their structure and location in the brain but also in their respective functional specificities. The neural network of the hippocampus plays an important role in the coding, storage, and retrieval of memory, whereas the cortex has a broader and more complex neural network, able to perform more complex information-processing tasks (37). Each brain region has its own unique structure and function, which together form the complex and sophisticated nervous system. Their interaction and coordination ensure the normal functioning of the brain, allowing engagement in complex neural activities such as thinking, perception, movement, and emotional expression (38).

#### 3.1.3 The dynamic regulatory mechanism of OCLN in the cerebrovascular system

The regulation of OCLN expression in the cerebrovascular system is highly environment-dependent, and the role of inflammatory factors and oxidative stress has been confirmed previously; however, the specific mechanisms and direction of effects vary significantly (39). Inflammatory factors such as cytokines and chemical mediators can affect the expression level of OCLN through multiple pathways. In an inflammatory response, these inflammatory factors can activate relevant signaling pathways, which can promote or inhibit the synthesis and release of OCLN. TNF-α significantly downregulates OCLN expression in cerebral microvascular endothelial cells through activation of the NF-κB signaling pathway, a phenomenon validated in an *in vitro* BBB model and an animal model of Alzheimer's disease (40). In contrast (Table 1), IL-6 transiently upregulated OCLN expression via the JAK-STAT3 pathway in the acute phase, whereas it shifted to an inhibitory effect in the chronic inflammatory state. This difference may be related to the negative feedback regulation of SOCS3 induced by the sustained activation of STAT3 (41). Oxidative stress is also an important regulatory mechanism that can affect the expression of OCLN by influencing the redox state in cells (33). Within cells, oxidative stress may lead to responses such as DNA damage, protein structural alterations, and loss of function, which can affect the transcription and translation processes of *OCLN* genes (Table 1). The regulation of OCLN by reactive oxygen species (ROS) clusters exhibits a concentration dependence. Low concentrations of ROS maintain basal OCLN expression through the Nrf2/ARE pathway, whereas high concentrations of ROS lead to reduced OCLN promoter activity by oxidative inactivation of the transcription factor Sp1. Notably, in ischemia–reperfusion injury models, NADPH oxidase–derived ROS were identified as the main causative agent of OCLN degradation (42). When inflammatory factors and oxidative stress act together, interactions among them may occur, including amplification or restraint, and coregulation of OCLN expression levels. There is cross-regulation between inflammation and oxidative stress For example, TNF-α induces ROS production, which in turn enhances NF-κB activation, forming a positive feedback loop. Microenvironmental factors such as hypoxia regulate OCLN expression in a HIF-1α-dependent manner. In a stroke model, acute hypoxia (6 h) upregulated OCLN to maintain barrier function, whereas sustained hypoxia (24 h) resulted in a significant decrease in expression (43). In addition, the effects of inflammatory factors and oxidative stress on the expression of OCLN may vary among different cell types and disease states (44). They influence the synthesis, release, and functioning of OCLN through a variety of pathways and interactions, thus playing important roles in a wide range of physiological and pathological processes. In addition, post-translational modifications play an important role in the expression and distribution of OCLN (45). For example, OCLN can undergo modification processes such as phosphorylation and glycosylation, which can change its function or positioning. Furthermore, microenvironmental factors such as oxygen, nutrients, and hormones affect the expression and distribution of OCLN. These microenvironmental factors regulate the synthesis, modification, and degradation of OCLN by interacting with cell surface receptors (45).

During the developmental stage of the cerebral vascular system, OCLN expression and distribution change dynamically (29). This dynamic change facilitates adaptation to the growth and remodeling processes of the vascular system. During aging, the expression and distribution of OCLN also change. The structure and function of the vascular system changes with age, resulting in corresponding adjustments in the expression level and distribution of OCLN (46). Therefore, understanding the regulatory mechanisms of OCLN expression and distribution in the cerebrovascular system is of great significance for studying the pathogenesis of cardiovascular and cerebrovascular diseases, developing preventive treatments, and delaying the aging process.

### 3.2 Distribution characteristics in the cardiovascular system

#### 3.2.1 Expression and barrier function in vascular endothelial cells

OCLN is prevalent in vascular endothelial cells and forms a rate-limiting transport structure in the cellular interstitium, which contains two extracellular loops that form a connective seal that plays an important role in maintaining vascular integrity and function. Its differential expression in the arterial, venous, and capillary endothelium is closely related to the barrier function of blood vessels (47). OCLN expression is particularly significant in the arterial endothelium. Because the arterial endothelium is subjected to high blood flow impact and pressure, a high expression of OCLN helps enhance the stability and integrity of the endothelium and maintain the mechanical barrier function of blood vessels. It also participates in signal transduction and intercellular interaction, promotes the proliferation and migration of arterial endothelial cells, and contributes to the repair and regeneration of arterial vessels (48). In contrast, OCLN expression in the venous endothelium is relatively low. The venous system is subjected to a lower blood pressure but must maintain a certain degree of permeability to achieve the exchange of materials between the blood and tissues. Therefore, OCLN in the venous endothelium plays an important role in maintaining vascular permeability and regulating the diastolic and contractile functions of blood vessels (49, 50). In the capillary endothelium, OCLN expression exhibits a unique pattern. The capillary endothelium is the main site at which blood exchanges substances with tissue fluid, and its barrier function is particularly important. A high expression of OCLN in the capillary endothelium helps form tight cellular connections, effectively preventing macromolecular substances in the blood from entering the tissue fluid and maintaining the balance of material exchange between blood and tissues. In addition, OCLN is involved in regulating the microenvironment of capillaries and plays an important role in maintaining the normal physiological function of tissues (48). In summary, differences in OCLN expression in the arteries, veins, and capillary endothelium are closely related to its important role in vascular barrier function. By regulating OCLN expression and function, the stability and permeability of blood vessels can be effectively maintained, and the normal flow of blood and exchange of substances between tissues can be ensured (47).

#### 3.2.2 Differences in OCLN distribution in various areas of the cardiovascular system

In the cardiovascular system, there are clear differences in OCLN distribution to various parts of the body. In major vessels such as the coronary arteries and aorta, the OCLN distribution is relatively dense, especially in the basal membrane region of vascular endothelial cells. This is because these major blood vessels undertake the important task of supplying blood to the heart and require strong stability and barrier functions to maintain their integrity and function (51).

In the coronary artery, OCLN distribution exhibits a network-like characteristic, which is closely connected to the vascular endothelial cells and helps form tight cellular connections, thereby maintaining the mechanical barrier function of blood vessels. This distribution helps protect the heart from harmful external factors and ensures normal functioning of the heart and blood supply. OCLN is more widely and evenly distributed in large blood vessels such as the aorta. These blood vessels must not only withstand high blood pressure but also maintain a certain degree of permeability to achieve material exchange between blood and tissue. Therefore, OCLN distribution at these sites helps strengthen the stability and permeability balance of blood vessels and maintain their normal function of blood vessels (52). In general, variations in the distribution of OCLN in different parts of the cardiovascular system are closely related to their physiological functions. This difference in distribution helps maintain the stability and permeability balance of blood vessels and ensures normal blood supply and heart function. In addition, OCLN distribution is also regulated by many factors, such as growth factors, and cytokines. The interactions and regulatory mechanisms of these factors in the cardiovascular system warrant further investigation.

#### 3.2.3 Regulation mechanism of expression

OCLN finely regulates the permeability of the blood vessel wall and blood flow velocity through its unique structure and function. In terms of hemodynamics, OCLN can affect the expansion and contraction of blood vessels, thereby regulating blood flow ability and pressure. In addition, it can closely bind to vascular endothelial cells, enhance the stability of the blood vessel wall, and thus affect the dynamic balance of blood flow (53). In terms of the regulation of vasoactive substances, OCLN interacts closely with growth factors such as vascular endothelial growth factor (VEGF) and Angiotensin II. VEGF promotes angiogenesis, and OCLN can enhance the stimulatory effect of VEGF on vascular endothelial cells through its receptor-mediated signaling pathway, thus promoting the formation of new blood vessels. Furthermore, OCLN can regulate the activity of Angiotensin II and other vasoconstrictive factors and maintain the balance of vascular tension by influencing the release and action of these factors (54–56). In summary, OCLN acts as a bridge and link in regulating hemodynamics and vasoactive substances, both of which influence vascular permeability and blood flow rate; OCLN also interacts with vasoactive substances to help maintain the normal function of the vasculature. Therefore, the study of OCLN will contribute to an in-depth understanding of the regulation mechanism of hemodynamics and provide new ideas and methods for the prevention and treatment of related diseases.

## 4 Dysfunctional OCLN mechanisms in cardiovascular and cerebrovascular diseases

### 4.1 Maintaining the structural integrity of the BBB

OCLN is an important component of cell connectivity and is widely present between epithelial and endothelial cells, especially in the BBB (8). The BBB is a special structure formed by tight ties between endothelial cells. Its integrity is crucial for protecting the nervous system (57). The BBB not only controls permeability allowing substances to enter and leave the brain, ensuring the stability of the brain environment, but also effectively protects the brain from the invasion of external harmful substances, bacteria, toxins, and pathogens and prevents macromolecular substances such as macromolecular proteins, cells, and other potentially harmful substances from entering the brain tissue to avoid damage to the nervous system (58, 59). OCLN directly affects the permeability and integrity of the BBB by regulating TJs between endothelial cells, which play a key role in maintaining physiological homeostasis in the brain (60).

In the BBB, multiple types of OCLN proteins synergize with the claudin-5/OCLN/ZO-1 complex to form a complex network of proteins that connect adjacent endothelial cells, resulting in a highly selective and tight barrier (61). The interactions of these OCLN proteins not only provide mechanical support and enhance the stability of cellular junctions but also regulate the passage of substances between cells by precisely regulating the size of cellular gaps, reducing the intercellular space, and thus forming a virtually impermeable barrier (62). This structure ensures that most substances in the blood, especially water-soluble small molecules and large proteins, cannot easily enter brain tissues, effectively preventing harmful substances from entering the brain and maintaining the normal function of the brain (63, 64).

OCLN stability and function directly determine BBB integrity. Specifically, members of the claudin family (e.g., claudin-3 and claudin-5) selectively allow certain small molecules and ions to pass through the BBB, while restricting the entry of larger or OCLN deleterious substances by regulating the permeability of intercellular TJs, its absence can lead to BBB leakage (65). OCLN mainly regulates the stability of cell membranes, maintains the structural function of TJs, and ensures the effectiveness of the BBB, which may compromise BBB integrity, resulting in increased BBB permeability and making it easier for harmful substances to enter the brain (66). ZO family proteins (e.g., ZO-1, ZO-2, and ZO-3) anchor OCLN to the actin cytoskeleton, further enhancing the function and localization of these OCLN proteins by interacting with the cytoskeleton, which contributes to the maintenance of the stability of the TJs between the BBB endothelial cells, and whose deletion results in the internalization of OCLN and (Table 1) disruption of the barrier. Furthermore, activation of the RhoA/ROCK pathway phosphorylates ZO-1, impairing its binding to OCLN and increasing BBB permeability (67). The role of ZO proteins is not limited to supporting the localization of proteins but also includes a synergistic role in the regulation of intercellular junctions, ensuring the stability of cell membranes and selective permeability of the BBB. Abnormalities in ZO proteins can lead to BBB dysfunction, thereby affecting the brain's ability to protect itself from harmful substances (10, 68). Therapeutic strategies targeting OCLN have become a hot research topic for restoring BBB function. OCLN stabilizers such as anti-inflammatory drugs (e.g., glucocorticoids) can reduce OCLN degradation by inflammatory factors by inhibiting the NF-κB pathway, and natural products (e.g., resveratrol) can enhance OCLN expression by activating the Nrf2 pathway (8). Moreover, HDAC inhibitors (e.g., sodium valproate) have been shown to restore OCLN expression and transcription through epigenetic regulation. In addition, in terms of miRNA regulation, miR-155 and miR-21 have been reported to promote the degradation of OCLN by targeting the 3'-UTR of OCLN, and the inhibition of these miRNAs can significantly enhance the levels of OCLN, whereas miR-132 can significantly enhance OCLN levels by inhibiting Rho-132. In contrast, one study found that miR-132 indirectly stabilized the OCLN–ZO-1 complex by inhibiting the RhoA/ROCK pathway (69). In addition, interventional strategies for BBB function restoration have shown potential in ischemic stroke models, where delivery of the *OCLN* gene via adeno-associated virus (AAV) has been shown to reduce brain edema and neuronal damage. Targeted activation of the Wnt/β-catenin pathway (e.g., lithium) upregulates OCLN expression and improves BBB leakage after cerebral hemorrhage. In the field of drug delivery and permeability modulation, nanoparticles [e.g., poly(lactic acid)-hydroxyacetic acid copolymer PLGA] are designed to release OCLN protectors in response to localized inflammatory microenvironments (e.g., MMP-9 overexpression), enabling precise repair (70).

### 4.2 Involvement in blood–brain barrier selective transport

OCLN plays an important role not only in maintaining barrier function but also in transporting specific substances (71). The BBB, as a barrier between the brain and the external environment, in addition to restricting the entry of harmful substances, requires precise regulation of the selective passage of small molecules such as water, ions, and nutrients to ensure the stability of the environment within the brain (72). By regulating the size and structure of cellular interstitial spaces, OCLN can control the passage of these small molecules in an orderly manner, thus maintaining BBB permeability and internal homeostasis in the brain (73). Some small molecules such as glucose and amino acids must enter the brain through specific transporter proteins, although there are certain limitations in the TJs of the BBB. OCLN is closely related to the function of these transport proteins and achieves selective transport by regulating the spatial distribution of transporter proteins. Specifically, by regulating the permeability of the BBB, OCLN provides the necessary “channels” and support for these transporter proteins, ensuring that needed small molecules can enter the brain efficiently and in an orderly manner, while limiting the penetration of harmful substances, For example, the OCLN/ZO-1 complex maintains the polar localization of the glucose transporter GLUT1 near TJs to ensure efficient glucose transport, and key regulation lies in the fact that activation of the Wnt/β-catenin pathway upregulates the expression of Claudin-5 and OCLN and enhances the ion-selective barrier (57).

In the BBB, different types of OCLN play various roles through their own mechanisms; for example, members of the claudin family, including Claudin-3 and Claudin-5, play a central role in regulating the permeability of the BBB (74, 75). These proteins control the passage of small molecules such as water and ions by selectively regulating the permeability of intercellular junctions. Different types of claudins have specific expression patterns in different tissues and can selectively allow certain substances to pass through the BBB, as needed (76). Claudin-5 is particularly important in the BBB, in which the tightness of the cellular gap directly regulates BBB function, restricting the entry of macromolecules such as endotoxins and pathogens into the blood vessels, while allowing the passage of important small molecules such as water and ions (77). In addition, OCLN proteins play a crucial role in BBB function. Although the exact mechanism of action is not fully understood, studies have shown that OCLN is essential for maintaining the permeability and stability of the BBB (78). OCLN proteins are involved in the formation and stabilization of TJs, ensuring the structural integrity of the BBB. When OCLN malfunctions, TJs in the BBB are compromised, leading to increased permeability, which, in turn, allows harmful substances to penetrate the brain, potentially causing neurological disorders (e.g., Alzheimer's disease and Parkinson's disease) (79–81).

In addition to claudins and OCLNs, ZO family proteins (e.g., ZO-1, ZO-2, and ZO-3) play an important role in regulating BBB permeability (67). ZO proteins regulate the TJs between endothelial cells of the BBB by interacting with claudins, OCLNs, and other OCLNs, thereby maintaining the selective permeability of the BBB. ZO proteins ensure the integrity and function of the BBB by stabilizing the TJs and by facilitating the attachment of OCLNs to the cytoskeleton. Once the ZO protein function is impaired, the selective permeability of the BBB is compromised, which may lead to pathological changes in the nervous system and the development of related diseases (82). The therapeutic strategies that can be adopted to address this pathophysiology are also worth mentioning. In OCLN-mediated transport regulation, FGF2 phosphorylates the tyrosine at position 408 of the OCLN through the FGFR1/PKCη pathway, which enhances the polar localization of GLUT1. In addition, nanocarrier technologies such as Angiopep-2 modified liposomes can realize brain-targeted drug delivery with the help of the dynamic open window of OCLN, for which more novel therapeutic tools can be investigated in the future (83).

### 4.3 Mediating dynamic regulation of the BBB

OCLN not only plays a crucial role in maintaining BBB structure and function but also dynamically regulates BBB permeability in response to changes in the external environment as well as stimuli from different pathologic states (84). The BBB is a protective barrier between the brain and the external environment. OCLN, as an important component, controls TJs between vascular endothelial cells and ensures the selective permeability of the BBB to prevent harmful substances and pathogens from entering the brain while maintaining the stability of the neural environment (85). However, when subjected to inflammatory responses, oxidative stress, certain drugs, or neurodegenerative diseases (e.g., Alzheimer's disease and Parkinson's disease), OCLN expression, structure, and function may be altered, leading to significant changes in BBB permeability (59, 81). Specifically, inflammatory factors such as tumor necrosis factor-α (TNF-α) and interleukin-1 (IL-1) can play a direct role in the degradation, conformational alteration, or repositioning of OCLN by activating several intracellular signaling pathways, particularly the NF-κB signaling pathway, which, in turn, induces the above changes by activating MMP-9, leading to relaxation of intercellular junctions and increased BBB permeability (86). These changes render the BBB ineffective in isolating harmful substances, making it easier for substances such as bacterial toxins, immune cells, and inflammatory factors to cross the BBB and enter the brain, potentially triggering or worsening inflammation and nerve damage in the brain. Also in the context of oxidative stress, ROS phosphorylate serine 490 of OCLN through the PKCδ pathway, thereby promoting its endocytosis (87).

OCLN dysfunction is closely associated with various neurological disorders, particularly chronic neurodegenerative diseases (73). In the cerebrovascular system of patients with Alzheimer's disease, Aβ deposition and downregulation of OCLN expression through the inhibition of the (Table 1) PI3 K/Akt pathway lead to increased BBB permeability (88). At this point, the accumulation of harmful substances such as β-amyloid is no longer effectively blocked, and these substances can penetrate the BBB, further aggravating neurodegenerative lesions and driving the disease process. In addition, BBB disruption may also lead to increased neuroinflammation, as immune cells and inflammatory factors can more easily enter the brain and interact with neurons, creating a vicious cycle that ultimately exacerbates brain damage (78, 89, 90). Furthermore, neuroinflammation itself often damages BBB integrity through direct or indirect pathways, and when the immune system is overactivated within the CNS, cytokines, chemokines, and other factors can trigger endothelial cell dysfunction, which, in turn, can lead to the degradation or remodeling of OCLN. This not only increases BBB permeability but may also lead to the entry of harmful substances such as free radicals and cytokines into brain tissues, causing a more extensive impairment of neurological function.

In cardiovascular and cerebrovascular diseases, downregulation of OCLN expression or dysfunction often leads to vascular endothelial barrier disruption, which can specifically manifest in the form of increased endothelial cell permeability, leading to a decrease in the barrier function of the vascular wall, easily triggering fluid leakage, plasma protein leakage, and other phenomena, which further promote local inflammatory reactions (30). It can also manifest as an increased inflammatory response in the vascular walls. Chronic inflammatory responses triggered by functional disruption of the vascular endothelium exacerbate the development of cardiovascular and cerebrovascular diseases; for example, the development of atherosclerosis and hypertension is closely associated with changes in the expression of OCLN, often accompanied by alterations in the function of the endothelium and the disruption of TJs. OCLN, as a component of TJs, regulates the barrier function between endothelial cells and maintains vascular permeability (91). Downregulation or dysfunction of OCLN expression is closely related to the development of atherosclerosis, and disruption of endothelial cell TJs leads to impaired vascular endothelial barrier function, which promotes the pathogenesis of cardiovascular diseases such as inflammatory response, lipid deposition, and thrombosis (92, 93). With regard to its therapeutic strategy, in inflammatory regulation, the TNF-α antibody infliximab reverses OCLN internalization by blocking NF-κB signaling. In addition, oxidative stress–targeted compounds such as edaravone can reduce the drop in OCLN phosphorylation levels by inhibiting the PKCδ/NADPH oxidase axis, and such compounds can be effectively applied in therapy.

### 4.4 Modulating inflammatory response

OCLNs also play an important role in the inflammatory response by regulating the barrier function of the vascular endothelium and influencing the migration and infiltration of inflammatory cells (e.g., leukocytes and monocytes) (94). In a healthy state, OCLN effectively restricts the infiltration and entry of inflammatory cells by maintaining tight intercellular junctions and limiting the permeability of macromolecules and cells between blood vessels and tissues, thus preventing overpenetration of inflammatory cells, whereas in an acute inflammatory response, impaired OCLN function leads to increased permeability of the blood vessel wall, which contributes to easier infiltration of inflammatory cells into the tissues. This phenomenon is usually caused by inflammatory mediators (e.g., histamine and leukotrienes) and inflammatory cells enter the damaged tissue through gaps between vascular endothelial cells. The infiltration of inflammatory cells exacerbates the local immune response and facilitates the progression of cardiovascular disease (95, 96).

It is worth mentioning that OCLN also plays a key role in the migration of immune cells, and the inflammatory response requires the rapid penetration of immune cells into the damaged area for an immune response (97). Integrin αVβ3 binds to the extracellular domain of OCLN during leukocyte migration and triggers Rac1/RhoA signaling to rearrange TJ. In this process, changes in OCLN promote the activation and chemotaxis of immune cells, thereby accelerating their infiltration. OCLN is also associated with the regulation of a variety of cytokines, particularly interacting with proinflammatory factors (e.g., TNF-α and IL-1β), and OCLN expression and function may be altered as a result. For example, cytokines such as TNF-α and IL-1 can lead to degradation or relocalization of OCLN. Such alterations can lead to loosening of intercellular junctions, which, in turn, allows inflammatory cells (e.g., neutrophils and macrophages) to more readily infiltrate tissues and trigger an inflammatory response. Moreover, during an inflammatory response, proinflammatory cytokines such as TNF-α and IL-1β are able to alter the structure of tight intercellular junctions by activating a variety of signaling pathways (94, 96). Specifically, TNF-α and IL-1β are able to inhibit OCLN promoter activity and reduce its transcription by activating the NF-κB signaling pathway, promoting OCLN degradation or dysfunction, which leads to the relaxation of intercellular junctions and increased vascular permeability (98).This alteration allows inflammatory cells to penetrate more easily into the site of inflammation, enhancing the local immune response and exacerbating the inflammatory process (99). In addition, inflammatory factors can further affect the barrier function of vascular endothelial cells by regulating the expression and localization of OCLN, thereby exacerbating tissue damage to some extent (100). OCLN plays an important role in signaling between the vascular endothelium and other cell types by participating in the formation and maintenance of intercellular TJs (101).

Cardiovascular diseases are usually accompanied by chronic inflammation and OCLN may play an important role in modulating inflammatory responses in cardiovascular diseases by regulating the integrity of the vascular barrier (102). For example, in patients with hypertension and atherosclerosis, TJ protein expression is often altered, and OCLN downregulation leads to increased permeability of the vascular wall, which promotes infiltration and deposition of inflammatory factors. The local inflammatory response is often further exacerbated by the loss of barrier function due to disruption of the OCLN proteins, not only aggravating the local inflammatory response but also potentially leading to tissue damage, creating a vicious cycle. In some chronic inflammatory diseases (e.g., rheumatoid arthritis and inflammatory bowel disease), OCLN alteration is often a key factor in pathology development (66). Therapeutic strategies targeting inflammation have also been investigated. In anti-inflammatory interventions, (Table 1) metformin inhibits MLCK-mediated OCLN cytophagy in an AMPK-dependent manner, and in addition, targeted delivery; for example, VCAM-1 antibody-coupled exosomes that can specifically deliver OCLN mRNA to the inflammatory endothelium can well intervene in the regulation of inflammation by OCLN (103).

### 4.5 Oxidative stress and apoptosis

Oxidative stress and apoptotic mechanisms are often concomitant in the pathogenesis of most cardiovascular diseases (104). Oxidative stress plays a very important role in cardiovascular disease; the main oxidative stress in cardiovascular injury is the response to ROS. ROS are a by-product of the normal metabolism of oxygen and play an important role in cell signaling and homeostasis *in vivo*. However, excessive amounts of ROS lead to oxidative stress, which, in turn, leads to vascular endothelial damage (105). Oxidative stress is involved in the pathogenesis of ischemic and hemorrhagic stroke and controls the permeability of the BBB to a certain extent, while excessive oxidative stress leads to apoptosis by damaging cellular macromolecules with oxygen free radicals (106). Apoptosis is a type of programmed cell death associated with various systemic diseases. It differs from necrosis and mainly manifests as reduced cell volume, concentrated chromatin, and fragmentation of the cell nucleus (107). Excessive apoptosis in the cardiovascular system leads to numerous adverse vascular outcomes such as cardiomyocyte and endothelial cell apoptosis, which are the main causes of myocardial dysfunction and vascular injury, respectively. Increased expression of some apoptotic proteins is observed during stroke, and inhibition of apoptosis protects against the development of some cardiovascular diseases (108).

Excessive oxidative stress leads to apoptosis, in which OCLN is degraded or its function is altered, thus affecting the barrier function of blood vessels. Oxidative stress affects the expression and function of OCLN (109, 110). Some evidence indicates that when OCLN is degraded by certain methods, it promotes the expression of apoptosis-related proteins, thereby promoting apoptosis in vascular endothelial cells, indirectly suggesting that OCLN is closely linked to apoptosis (111). In cardiovascular and cerebrovascular system diseases, a variety of classical signaling pathways can regulate OCLN protein expression and distribution to participate in apoptotic processes that damage the cardiovascular and cerebrovascular systems; examples of these pathways are protein kinase C (PKC), MAPK, phosphatidylinositol 3-kinase (PI3K)/Akt and Wnt/β-catenin, and other pathways (18). When oxidative stress activates the p38 MAPK pathway, phosphorylated OCLN promotes its ubiquitinated degradation, whereas when activated by OCLN, deletion in the BAX/BAK pathway induces endothelial cell apoptosis. Notably, the antioxidant NAC can maintain OCLN expression through the Nrf2 pathway (112). However, some studies have found that OCLN can act as upstream regulatory molecules to affect signaling pathways. For example, in human cerebral microvascular endothelial cells, lowering OCLN can upregulate the PI3K/AKT and ERK signaling pathways and promote apoptotic protein expression, which can lead to the development of cardiovascular diseases (113). Although there is no direct evidence that OCLN is directly related to oxidative stress and apoptosis in cardiovascular diseases, it can be indirectly illustrated from a series of studies that OCLN is closely related to oxidative stress and apoptosis (114). Antioxidants such as melatonin (Table 1) can inhibit OCLN ubiquitination degradation through the SIRT3/FoxO3a pathway. In gene therapy, AAV9-mediated overexpression of OCLN can inhibit the BAX/BAK pathway to reduce endothelial apoptosis. Although there are a few studies on the oxidative stress and apoptosis of OCLN in the cardiovascular system, further exploration of the specific mechanisms of OCLN in the cardiovascular system regarding oxidative stress and apoptosis can provide new ideas and methods for the treatment of related cardiovascular diseases.

### 4.6 Vascular remodeling

Vascular remodeling is a complex physiological process that involves structural changes in the vascular wall, often involving proliferation and migration of vascular wall cells, endothelial dysfunction, and synthesis and degradation of the extracellular matrix (115). There are two contrasting forms of vascular remodeling: physiologic vascular remodeling occurs primarily because of blood supply demands or organ growth and development, whereas pathologic vascular remodeling occurs primarily because of inadequate blood supply or tissue damage (116). Pathological vascular remodeling plays a very important role in the development and progression of cardiovascular diseases and can be observed in hypertension, atherosclerosis, aortic aneurysms, and restenosis. The extent of vascular remodeling is reflected in changes in medial thickness, lumen diameter, transverse zones, and their ratios (117). Vascular remodeling varies in different physiological situations; dystrophic remodeling results in a thinning of the vessel wall and a decrease in the wall-to-lumen ratio, whereas hypertrophic remodeling results in a thickening of the vessel wall and an increase in the wall-to-lumen ratio. In eutrophication, the wall-to-lumen ratio is not altered, although the size of the vessel may vary. Depending on the situation, different types of vascular remodeling will occur (118). Furthermore, vascular remodeling plays an important role in the growth and metastasis of tumors of the cardiovascular system. Tumor growth and development depend on adequate blood supply of oxygen and nutrients, and tumor cells release substances that promote blood vessel formation. Vascular remodeling provides tumors with the necessary blood supply and nutrients, leading to further growth and spread (119). Pathological vascular remodeling usually occurs under stimulating conditions such as inflammation and trauma. Oxidative stress and inflammation are key factors in endothelial dysfunction during pathological vascular remodeling. It is generally believed that the pathogenesis of vascular remodeling begins with endothelial cell dysfunction; thus, pathological vascular remodeling is closely related to oxidative stress and inflammatory response (120).

OCLN, an important component of TJs, helps maintain the stability of vascular endothelial barrier function, thereby further affecting vascular remodeling to a certain extent (121). OCLN is involved in inducing cell migration and may be involved in regulating processes such as migration of vascular smooth muscle, which further affects vascular remodeling. For example, VEGF regulates endothelial cell migration by phosphorylating OCLN S490 via PKCδ (122). In addition, OCLN can be involved in pathological remodeling, with angiotensin II promoting vascular fibrosis by downregulating OCLN through the AT1 receptor, whereas some studies have found that OCLN overexpression inhibits the TGF-β/Smad2 pathway, thereby attenuating vascular wall thickening, with targeting potential (123). Some studies have also found that OCLN can be regulated by the corresponding signaling pathway, before regulating it. For example, VEGF induces the phosphorylation of OCLN at the S490 locus. OCLN S490 phosphorylation induces the expression of VEGF in endothelial cells, which promotes the proliferation of these cells, as well as the regeneration of blood vessels, thus preventing the occurrence of diseases of the cardiocerebral vascular system (124). Although there are a few direct studies on the role of OCLN in vascular remodeling in the cardiovascular system, its role in maintaining vascular endothelial barrier function, regulating cell signaling, and oxidative stress suggests a potential link between OCLN and the process of vascular remodeling (Table 1). Notably, in terms of vasoprotection, rapamycin inhibits OCLN internalization to improve revascularization through the mTORC2/RhoA pathway, and in addition, targets modulations such as siRNA silencing of AT1R-reversed AngII-induced OCLN downregulation. Hence, further understanding of the role of OCLN in the process of vascular remodeling may provide new ideas and methods for the prevention and treatment of diseases of the cardiovascular and cerebrovascular system.

## 5 Diagnosis and treatment of OCLN and cardiovascular diseases

### 5.1 Atherosclerosis

Atherosclerosis is a common chronic vascular disease characterized by lipid accumulation, fibrous tissue proliferation, and calcium deposits in the arterial wall, leading to the thickening and hardening of the arterial lumen and narrowing or occlusion of the arterial wall (125, 126). This process involves a variety of cellular and molecular processes, with aberrant expression and dysfunction of OCLN thought to be important in promoting the development of atherosclerosis (127). As a protein closely related to vascular endothelial cells, OCLN has received much attention in the diagnosis and treatment of atherosclerotic diseases in recent years (47, 128). In vascular diseases such as atherosclerosis, the expression and functional status of OCLN are altered. Because of their involvement in the maintenance of intercellular barriers and cell polarity, the abnormal OCLN expression and function lead to dysfunction of the endothelial barrier and promote inflammatory cell infiltration and lipid deposition, accelerating the formation and development of atherosclerotic plaques (4). In addition, downregulated expression or impaired function of OCLN leads to loosening of endothelial cell-to-cell junctions and increased vascular permeability, making it easier for blood lipids, inflammatory cells, and cytokines to penetrate the vessel wall and promote the formation of atherosclerotic plaques (129). OCLN can also affect the expression and release of inflammatory factors by regulating signaling pathways such as NF-κB and MAPK and can inhibit apoptosis by regulating signaling pathways such as PI3 K/Akt and Bcl-2 (130). Moreover, downregulated expression of OCLN promotes apoptosis in endothelial and smooth muscle cells, leading to plaque destabilization and increased risk of plaque rupture, promoting inflammatory responses, and exacerbating inflammatory damage in atherosclerosis (131). In addition, OCLN inhibits NADPH oxidase activity and reduces ROS production; downregulation of its expression leads to increased oxidative stress, which damages endothelial cells and promotes atherosclerosis development (132).

This evidence indicates that OCLN expression level is negatively correlated with the severity of atherosclerosis, suggesting that it plays a contributing role in the development of atherosclerosis (133). Recently, an increasing number of studies have used OCLN as a biomarker of atherosclerosis, based on its expression levels and functional status; the risk of atherosclerosis can be assessed by measuring the blood levels of OCLN (53). Some studies have shown that OCLN justifies being a novel biomarker of AS in the sense that serum OCLN level is negatively correlated with AS plaque stability, and the combined detection of OCLN and hs-CRP can improve the detection rate of early AS. In addition, molecular imaging of atherosclerotic plaques can be performed using molecular probes targeting OCLN to improve the accuracy and specificity of atherosclerosis diagnosis (134). Furthermore, the detection of OCLN can be combined with other biomarkers and clinical indicators to aid diagnosis (55). Intervention strategies for OCLN have been studied to provide new ideas for the treatment of atherosclerosis. Some medications (e.g., statins and aspirin) and natural products (e.g., resveratrol and curcumin) can upregulate the expression of OCLN, improve endothelial function, and inhibit the development of atherosclerosis (135, 136). In gene therapy, AAV9-mediated overexpression of OCLN significantly improves endothelial function and reduces macrophage infiltration in plaques in ApoE−/− mice. Some antioxidants and anti-inflammatory drugs can protect OCLN from damage caused by oxidative stress and inflammatory factors and maintain its normal function. It is worth mentioning that by utilizing OCLN as a target, medications can be delivered precisely to the atherosclerotic plaque site, improving their therapeutic effect and reducing side effects (137). Moreover, liposomes modified with OCLN antibodies can specifically deliver statins to plaque sites, and, in animal experiments, lead to a reduction in plaque load. The level of OCLN expression is closely related to the prognosis of patients with atherosclerosis; downregulation of OCLN expression leads to plaque instability and increases the risk of plaque rupture and thrombosis, thereby increasing the incidence of myocardial infarction and stroke (138). Patients with atherosclerosis and low serum OCLN levels have a higher risk of cardiovascular events (139).

The dysfunctional targeting of OCLN can also be regulated pharmacologically to regulate its expression and function (140, 141). For example, medications are used to inhibit the overexpression of OCLN or promote its normal expression and function, thereby slowing the process of atherosclerosis. Gene therapy is an emerging therapeutic approach that can be used to treat diseases by altering the expression of the patient's genes through genetic engineering means (142). By introducing vectors containing specific genes, patients can express normal OCLN, thereby improving the function of vascular endothelial cells and reducing the progression of atherosclerosis (143). It is also possible to treat atherosclerosis through gene editing technology, which can enable the modification or replacement of the gene for OCLN, which has potential therapeutic benefits; however, further research and clinical trials are needed to validate this (144). For patients with severe atherosclerosis, surgical treatment may still be necessary. For example, angioplasty or stenting is used to restore the patency of blood vessels. During the surgical procedure, the surgical effect and prognosis can be assessed by testing OCLN expression and function. In conclusion, OCLN is valuable in the diagnosis and treatment of atherosclerosis. Assessing OCLN expression and function can assist in evaluating the risk and severity of atherosclerosis, providing a basis for early diagnosis and risk assessment. A variety of methods such as drug therapy, gene therapy, lifestyle intervention, and surgical treatment can be used to treat OCLN dysfunction. In-depth research on atresia OCLN suggests that more therapeutic methods will be applied to the clinical treatment of atherosclerosis in the future.

### 5.2 Ischemic cerebrovascular disease

Ischemic cerebrovascular disease refers to a series of neurological impairments and dysfunctions caused by insufficient blood supply to the brain due to narrowing or occlusion of blood vessels in the brain (145). Some of the most common conditions that can be treated are transient ischemic attack (TIA), cerebral thrombosis, and cerebral infarction, which are ischemic conditions in the brain caused by a lack of blood supply (146). With recent advancements in medical research, the role of OCLN as an important extracellular matrix protein in vascular regeneration and repair has been gradually recognized; OCLN can be used as a biomarker to help clinicians determine whether a patient is in the early stages of TIA so that they can provide timely intervention measures (147). TIA can be more accurately diagnosed in combination with other imaging tests and clinical manifestations.

**Figure 3:**
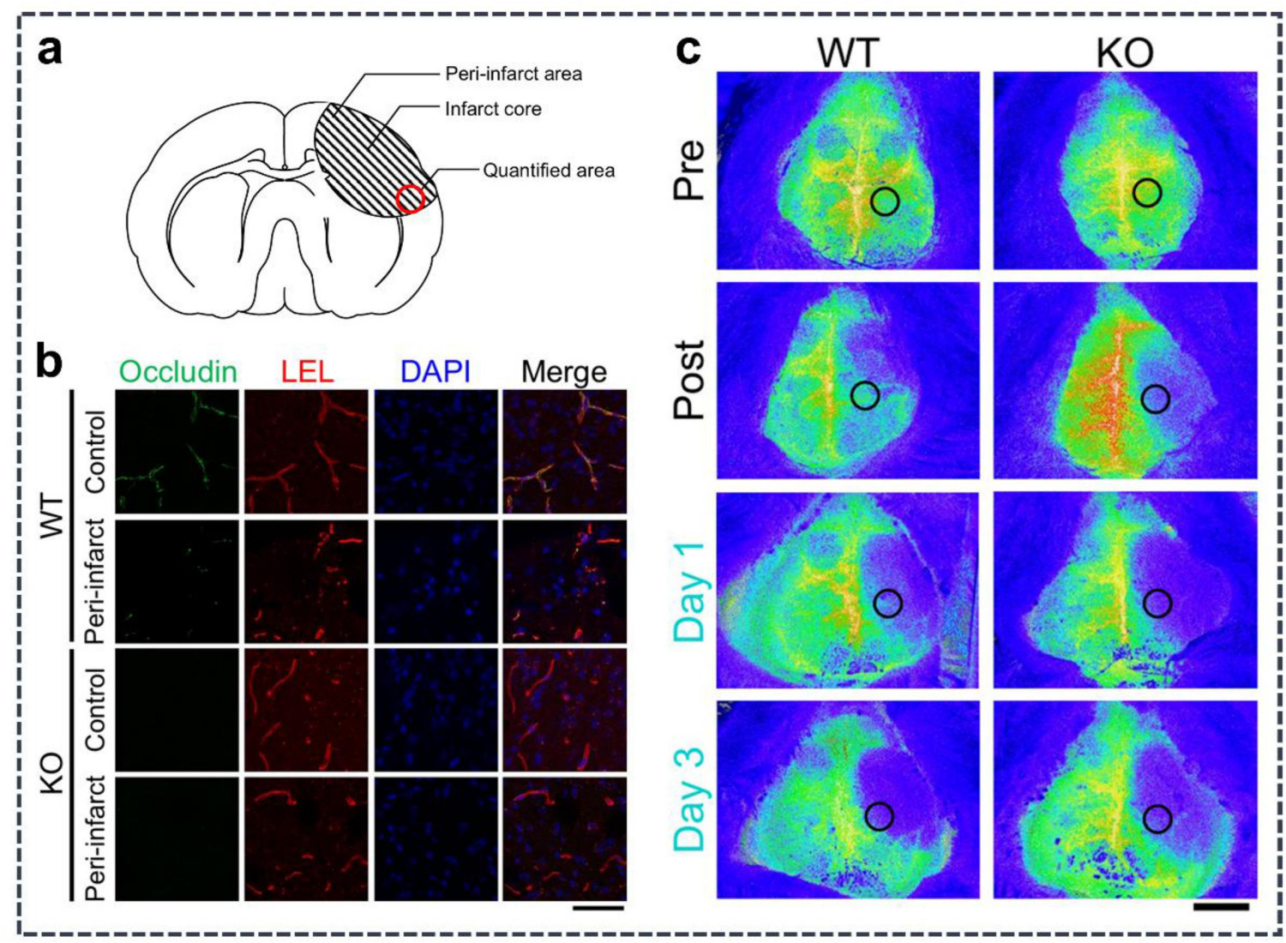
Occludin regulates BBB integrity and neurological function after stroke in mice. **(a)** Brain infarct zoning after ischemic stroke. **(b)** Immunofluorescence of occludin in different infarct zones. **(c)** Cerebral perfusion in wild-type and occludin-deficient mice after stroke. Adapted with permission from (60).

In ischemic cerebrovascular disease, aberrant OCLN expression and function lead to BBB disruption and promote inflammatory responses and neuronal injury, thereby exacerbating cerebral ischemia–reperfusion injury (60, 148) (Figure 3). After cerebral ischemia, downregulated expression or impaired function of OCLN leads to loosening of endothelial cell-to-cell connections, increasing BBB permeability, making it easier for harmful substances in the blood to penetrate brain tissues and exacerbating brain damage. Similarly, OCLN can affect the expression and release of inflammatory factors by regulating signaling pathways such as NF-κB and MAPK (8). Downregulation of OCLN expression also promotes inflammatory responses and exacerbates cerebral ischemia–reperfusion injury (149). When cerebral ischemia is followed by reperfusion, a large amount of reactive oxygen species is produced, leading to oxidative stress injury, at which point OCLN comes into play, inhibiting the activity of oxidative enzymes and reducing the production of reactive oxygen species. Therefore, downregulation of OCLN expression leads to increased levels of oxidative stress and damage to neurons and vascular endothelial cells. OCLN can also inhibit apoptosis by regulating PI3K/Akt, Bcl-2, and other signaling pathways; downregulation of its expression can promote apoptosis of neurons and endothelial cells, exacerbating brain damage (93, 134). The main pathological mechanism of ischemic cerebrovascular disease is insufficient blood supply to the brain, resulting in hypoxia and neuronal damage to brain tissue (150). Therefore, promoting vascular regeneration is one of the most important means of treating ischemic cerebrovascular disease, as it promotes the proliferation and migration of vascular smooth muscle cells, thereby promoting the formation and maturation of blood vessels (8). In ischemic cerebrovascular disease, vascular regeneration in ischemic regions can be promoted by the administration of exogenous OCLN or by pharmacological modulation of endogenous OCLN expression, which improves cerebral blood supply and attenuates brain tissue injury (151). Ischemic cerebrovascular disease leads to hypoxia and neuronal damage in brain tissue, which, in turn, triggers a series of neurological dysfunctions. As an important neuroprotective molecule, OCLN not only promotes vascular regeneration but also protects neurons from ischemic and hypoxic damage by regulating neuronal growth and differentiation, which is involved in maintaining BBB integrity and can play an important protective role in ischemic and other pathological conditions (152). In TIA disorders, OCLN levels may change; therefore, OCLN levels and activity can be assayed to help diagnose TIA (60). In ischemic cerebrovascular disease, administration of OCLN can reduce the extent of neuronal damage and promote the recovery of neurological function by increasing the content or activity of OCLN as a means of reducing ischemic damage to brain tissue and promoting neuronal recovery (153). Recently, strategies for treating ischemic cerebrovascular disease by pharmacologically modulating OCLN expression or function have been increasingly explored (154). For example, edaravone, a potent free radical scavenger, widely used in the treatment of stroke, not only attenuates oxidative stress but also protects BBB integrity by upregulating OCLN expression. Edaravone significantly increases the expression level of OCLN in the brain tissues of rats with cerebral ischemia and reduces BBB permeability, thus alleviating cerebral edema and neuronal damage (155). Edaravone reduces the volume of cerebral infarction by upregulating OCLN expression by a mechanism related to the inhibition of MMP-9-mediated OCLN degradation. Resveratrol, a natural polyphenolic compound with antioxidant, anti-inflammatory, and neuroprotective effects, is able to upregulate the expression of OCLN by activating the PI3 K/Akt signaling pathway, thereby protecting the integrity of the BBB and alleviating cerebral ischemia/reperfusion injury. The experimental results of a study showed that resveratrol pretreatment could significantly decrease the volume of cerebral infarction and reduce neurological deficits in a mouse model of cerebral ischemia. Resveratrol activation of the SIRT1/OCLN pathway resulted in improved neurological function scores after reperfusion (156). Nimodipine, a calcium channel blocker, is commonly used to treat cerebral vasospasm, and it not only improves cerebral blood flow but also protects the BBB by upregulating OCLN expression. In one study, it was found that nimodipine treatment significantly reduced BBB permeability and decreased inflammatory factor levels in the brain tissue of rats with cerebral ischemia (157). Curcumin is a natural compound extracted from turmeric with anti-inflammatory, antioxidant, and neuroprotective effects. Recent evidence indicates that curcumin can reduce cerebral ischemia–reperfusion injury by inhibiting the NF-κB signaling pathway to upregulate the expression of OCLN. Curcumin treatment can significantly reduce brain edema and neuronal apoptosis in mice modeled with cerebral ischemia (158).

After the occurrence of ischemic cerebrovascular disease, the body experiences a series of inflammatory reactions, leading to further damage to brain tissue. OCLN can reduce the inflammatory damage of brain tissue by inhibiting the inflammatory response. OCLN can also regulate the activation and migration of immune cells, thus inhibiting the occurrence and development of inflammatory reactions. In addition, some investigators have made progress in bioengineering therapeutic strategies, with collagen scaffolds loaded with OCLN overexpressing MSCs showing improved vessel density and increased synaptic regeneration in stroke models. In conclusion, OCLN has an important role in the treatment of ischemic cerebrovascular disease. Through various mechanisms such as promoting vascular regeneration, protecting neurons, and inhibiting inflammatory response, OCLN can effectively improve the blood supply of ischemic brain tissue, reduce brain tissue injury, and promote neurological function recovery. Although the use of OCLN is still undergoing research and clinical trials, further technological and medical advancements are likely to reveal OCLN as an important means of treating ischemic cerebrovascular diseases in the future.

### 5.3 Cerebral vascular stenosis and occlusion

Cerebrovascular stenosis, a common neurological disorder, refers to stenosis or occlusion of the blood vessels in the brain due to arteriosclerosis, arterial inflammation, or fibrous muscle dysplasia, resulting in insufficient blood supply in the brain or blocked blood flow (159). Cerebrovascular occlusive disease, on the other hand, is a series of disorders caused by the occlusion of blood vessels in the brain due to various disease conditions such as thrombosis, atherosclerosis, vascular inflammation, and vascular spasm, resulting in the blockage of blood supply to the brain, which, in turn, affects the normal function and metabolism of brain cells (160). OCLN can serve as an important suggestive substance, and its expression and function may be abnormal in cerebrovascular stenosis and occlusion diseases. Dynamic changes in OCLN can reflect the extent of vascular remodeling, as it is closely related to disease pathogenesis and progression. Cerebrovascular stenosis and occlusion are often accompanied by damage to vascular endothelial cells and disruption of the BBB. OCLN expression levels can reflect the integrity of vascular endothelial cells and the permeability of the BBB, and OCLN maintains the stability of blood vessels by interacting with vascular endothelial cells and other cellular components.

In normal blood vessels, OCLN interacts with other extracellular matrix components to maintain vascular stability; however, this stability may be disrupted during cerebral vascular stenosis and occlusion (161). OCLN can interact with vascular endothelial and smooth muscle cells to help maintain the structural integrity of blood vessels by promoting cell proliferation and migration, which can assist in repairing damaged vessel walls, thereby slowing the progression of stenosis (162). OCLN plays an important role in regulating the inflammatory response. Cerebral vascular stenosis and occlusion are often accompanied by an inflammatory response that further exacerbates vascular damage. OCLN can inhibit the release of inflammatory factors and reduce the damage of inflammation on blood vessels (163), promote the production of anti-inflammatory factors, and accelerate the reduction of inflammation. By regulating the inflammatory response, OCLN helps reduce vascular damage and promotes vascular repair and regeneration. OCLN is involved in the process of signaling and cell interaction and can transmit signals by interacting with other cell surface receptors to influence processes such as cell growth, migration, and differentiation. In cerebrovascular diseases, OCLN may be associated with the proliferation and migration of vascular smooth muscle cells. By regulating smooth muscle cell activity, OCLN can influence the extent and progression of vascular stenosis (164). OCLN also interacts with other growth factors and cytokines to regulate blood vessel growth and development and plays an important role in the maintenance of the BBB. In cases of cerebral vascular stenosis and occlusion, the integrity of the BBB may be compromised, making brain tissue more susceptible to damage. OCLN can enhance the stability of the BBB and reduce the penetration of harmful substances, thus protecting brain tissue from damage (164, 165).

Recent intervention strategies targeting OCLN have provided novel ideas for treating cerebrovascular stenosis and occlusion. Edalavone could significantly increase OCLN expression in the brain tissues of rats in a cerebral ischemia model, reduce the permeability of the BBB, and minimize cerebral edema and neuronal damage. Resveratrol could upregulate OCLN expression by activating the PI3 K/Akt signaling pathway, thereby protecting the integrity of the BBB and reducing cerebral ischemia and reperfusion injury (166). Similarly, a number of other antioxidants and anti-inflammatory agents protect OCLN from damage caused by oxidative stress and inflammatory factors and maintain its normal function. By utilizing OCLN as a target, medications can be delivered precisely to the site of cerebral ischemia, improving the therapeutic effect and reducing side effects (167). PET-CT using [68Ga]-labeled OCLN-targeted probes can show specific uptake at stenosis sites. By examining the expression levels of OCLN in the blood or other body fluids of patients, it is also possible to assess the extent of cerebrovascular occlusive disease and its prognosis. In cases of OCLN dysfunction, its expression and function can also be regulated by surgical treatment or gene editing techniques. In addition, low-intensity pulsed ultrasound significantly improves collateral circulation by upregulating OCLN expression through mechanical force stimulation, which is not an ineffective intervention strategy (168). In conclusion, OCLN plays important diagnostic and therapeutic roles in cerebrovascular stenotic diseases. An in-depth study of the biological properties and mechanism of action of OCLN can elucidate the pathogenesis and developmental process of these diseases and provide new ideas and methods for their diagnosis and treatment. Future research on OCLN is anticipated to provide improved benefits and hope for patients.

### 5.4 Alzheimer's disease

Alzheimer's disease is a chronic neurodegenerative disease characterized by a progressive decline in cognitive function. Its pathogenesis is complex, involving neuronal damage, abnormal inter-neuronal connections, neurotransmitter imbalance, inflammatory response, etc. (169). Among them, abnormal intraneuronal connectivity is considered an important mechanism in the pathogenesis of AD. OCLN, as an important molecule in maintaining the BBB and intraneuronal connectivity, may play an important role in the pathogenesis of AD. Aβ42 induces aberrant phosphorylation of OCLN (Thr404 site), leading to BBB leakage. Some studies have found that the expression level of OCLN is significantly higher in the brains of patients with AD and vascular dementia than in normal senescent controls, especially in the frontal cortex and basal ganglia regions of the brain (170). OCLN is mainly expressed in pyramidal neurons responsible for cognitive processes, which are also the cell types affected by AD pathology. This finding may have important implications for understanding new pathogenic mechanisms of dementia and the novel role of OCLN and TJs in the disease (171). Abnormalities in intraneuronal connectivity in the brains of patients with AD may lead to increased BBB permeability, which, in turn, affects the expression and distribution of OCLN. Therefore, detecting the expression level and distribution of OCLN may provide new clues for AD diagnosis.

OCLN is an important component of the BBB, and changes in its expression levels reflect BBB permeability (172). By detecting the expression of OCLN, the degree of BBB impairment in patients with AD can be indirectly assessed for diagnostic purposes (173). OCLN expression in brain tissues or body fluids of patients may provide information regarding BBB permeability and the status of intraneuronal connectivity, thus providing a reference for the diagnosis of AD (170). Similarly, changes in OCLN expression may serve as biomarkers for early diagnosis and monitoring of AD and treatment effectiveness (174). AD can be more accurately diagnosed in combination with other biomarkers and imaging tests. Treatment of AD is currently based on a combination of pharmacological and non-pharmacological interventions (175). Some evidence indicates that certain medications can significantly increase the expression level of OCLN in the brain tissue of AD model mice and reduce BBB permeability, which, in turn, reduce neuroinflammation and neuronal damage and upregulate OCLN expression by activating the PI3 K/Akt signaling pathway. This protects the integrity of the BBB, reducing neuroinflammation and neuronal damage and improving cognition in AD model mice (166, 167). With regard to non-pharmacological treatments, one study reported that regular aerobic exercise showed potential in upregulating OCLN expression in the brain tissue of AD model mice, attenuating neuroinflammation and neuronal damage and improving cognitive function (176). In addition, cognitive training can upregulate OCLN expression by activating the brain-derived neurotrophic factor signaling pathway, which can also improve the cognitive function of patients with AD (177). Gene therapy can also be effective in improving the cognitive function of mice with AD. This was proved when an AAV vector was used to deliver the *OCLN* gene to the brains of AD model mice and gene therapy was also effective in ameliorating AD symptoms. A nanoparticle-based drug delivery system that can deliver OCLN agonists to the brain of mice with AD has been developed, and it has demonstrated that upregulation of OCLN levels can improve the cognitive function of patients with AD. siRNA nanoparticles targeting OCLN increase the efficiency of Tau protein translocation across the BBB by several fold (178). Photobiomodulation therapy (630 nm) can improve cognitive scores in patients with AD by activating the TRPV4–OCLN axis.

However, because of the complex pathogenesis of AD, extant treatments can only relieve symptoms and cannot fundamentally cure the disease. However, the latest neurosurgical treatment of AD has provided research directions and hope. OCLN has potential applications in the treatment of AD (179). Furthermore, regulating OCLN expression and distribution can strengthen BBB permeability, reducing the entry of harmful substances and signals into the brain and thus minimizing neuronal damage. In addition, abnormal OCLN expression may be related to abnormality in inter-neuronal connections, and regulating OCLN function can strengthen inter-neuronal connections and promote reconstruction of neural networks. In conclusion, OCLN has great value in the diagnosis and treatment of Alzheimer's disease. Clarification of the biological properties of OCLN and the mechanism of action in AD can provide insights into the diagnosis of AD and new targets and strategies for the treatment of AD. Future research should explore the therapeutic approaches for AD targeting OCLN by combining cutting-edge technologies such as gene editing technology and cell therapy, with the aim of providing more choices and hope for the treatment of AD. It is also necessary to strengthen efforts for early screening for AD so that preventive measures can be taken, increase public awareness about the disease, and make collaborative efforts to overcome this global challenge.

### 5.5 Myocardial infarction

Myocardial infarction (MI) is an adverse cardiovascular event caused by the formation of plaque or blood clots in the lining of the coronary arteries that impede the flow of blood to the heart and damage the myocardium, resulting from a lack of oxygen (180). The most common cause of myocardial infarction is atherosclerosis of the coronary arteries. When plaque ruptures or erodes, it can trigger platelet aggregation and thrombosis, further exacerbating stenosis or complete occlusion of the vessel. Patients with thin coronary arteries are more likely to have restricted blood flow. When the formation of a thrombus reaches a certain point, it leads to complete blockage of the coronary arteries, and the supply of blood to the myocardium is disrupted, leaving the myocardium in a state of ischemia and hypoxia, finally leading to myocardial necrosis (181). MI is characterized by a necrosis of cardiomyocytes and acute loss of myocardial tissue, resulting in structural and biomechanical changes to preserve cardiac function and minimize diastolic and systolic wall stress; these changes include collagen deposition with scar formation, fibrosis, hypertrophy, and alterations in the ventricular structure, leading to ventricular remodeling (182).

In the initial phase of MI, coronary artery obstruction leads to localized ischemia and hypoxia in myocardial tissues, which, in turn, leads to intracellular disturbances in cardiomyocytes, such as ATP depletion, calcium overload, and mitochondrial dysfunction, resulting in a pathological increase in ROS levels. This increase can lead to a series of adverse effects, including apoptosis and subsequent inflammatory responses (183). ROS can affect OCLN expression through a number of routes, including activation of NF-kB and MAPK signaling pathways, whereas OCLN is important for intracellular signaling to maintain autophagy, and downregulation of its expression can exacerbate autophagic flow impairment (184). In turn, necrotic tissue and degraded extracellular matrix induce aseptic inflammation involving molecular cascades and cellular responses. In contrast, OCLN can affect inflammatory cell infiltration and inflammatory factor release to some extent by regulating tight junction integrity (185, 186). In addition, OCLN affects the activation and transdifferentiation of myofibroblasts by modulating the TGF-β/Smad signaling pathway, which regulates the balance between collagen synthesis and degradation and consequently influences the course of myocardial fibrosis and ventricular remodeling. This further explains why OCLN delays the onset and progression of myocardial infarction by acting on myofibroblasts (187). OCLN is valuable in therapeutic studies of myocardial infarction, for example, in the treatment of coronary microcirculatory dysfunction after myocardial infarction with trimetazidine. Activation of AMPK signaling-related proteins, including OCLN, by trimetazidine could reduce coronary microcirculatory dysfunction after myocardial infarction and, thus, further reduce myocardial damage and improve prognosis (188). Other evidence also indicates that decreasing cell permeability by increasing OCLN expression allows α1-antitrypsin to exert a cytoprotective effect in vascular endothelial cells in the presence of hypoxia, further suggesting that OCLN could be a potential target for myocardial infarction therapy (189). Recent evidence indicates that OCLN plays a protective role in myocardial ischemia–reperfusion injury by modulating the Nrf2/HO-1 signaling pathway to attenuate oxidative stress injury. OCLN is also used as a diagnostic and prognostic indicator for myocardial infarction. For example, with regard to the protective effect of intestinal flora and metabolites in regulating miR-30a-5p in myocardial infarction, a comparative study on OCLN expression can further show that the imbalance of intestinal flora is closely related to myocardial infarction (190). Recent clinical studies have shown that plasma OCLN levels are significantly associated with left ventricular function in patients with myocardial infarction. When plasma OCLN concentration was lower than 8.2 ng/mL, patients had a 4.7-fold increased risk of adverse left ventricular remodeling (OR = 4.7, *p* = 0.003), a threshold that is an important guide for early risk stratification and intervention. Imaging studies revealed that OCLN expression levels were significantly correlated with infarct area and myocardial salvage index assessed by cardiac magnetic resonance imaging, suggesting that OCLN can be used as an adjunctive index for non-invasive assessment of the extent of myocardial injury (191). In terms of myocardial repair, OCLN shows significant therapeutic potential. Experimental studies demonstrated that exosomally delivered OCLN mRNA significantly reduced apoptosis in the infarct border zone (quantitatively verified by TUNEL staining), and that this protective effect was associated with the maintenance of intercellular junction integrity, attenuation of oxidative stress injury, and improvement of energy metabolism by OCLN. Gene therapy studies have also confirmed that overexpression of OCLN by adeno-associated virus vectors significantly improves cardiac function after myocardial infarction, reduces fibrotic area, and promotes neovascularization. In addition, OCLN-derived peptides (e.g., OCLN-20) have been developed as novel therapeutic agents that show potential to attenuate myocardial ischemia–reperfusion injury in animal models. In conclusion, OCLN plays a critical role in the pathologic process, diagnosis, treatment, and prognostic assessment of myocardial infarction (192). Elucidating the biological properties of OCLN and the mechanism of action in myocardial infarction can provide new clues for the diagnosis of myocardial infarction and new targets and strategies for the treatment of myocardial infarction. Future studies should further explore the key molecules in the OCLN regulatory network, develop precision therapeutics targeting OCLN, and optimize their application in the clinic. It is also necessary to undertake early screening to prevent myocardial infarction, raise public awareness of this condition, and make collaborative efforts to overcome this global phenomenon.

### 5.6 Heart failure

Heart failure (HF) is a complex clinical syndrome and a common end stage of many heart diseases (193). HF is characterized by poor oxygen delivery, fatigue with light physical activity (NYHA Classification), fluid retention (jugular varicose vein, lower extremity edema), shortness of breath, and impaired function of the heart itself, as evidenced by decreased diastolic or systolic function (194). Heart failure involves a complex and diverse set of pathologic mechanisms, including multiple effects of some compensatory mechanisms in different situations, such as neurohumoral activation [renin–angiotensin–aldosterone system (RAAS) system, sympathetic nervous system], alterations in cellular structure and function, subcellular molecular abnormalities, and disturbed cellular interactions. It also entails remodeling of the myocardium, including inflammation and endothelial dysfunction, cardiomyocyte hypertrophy and death, and altered myocardial tone and fibers. In addition, some systemic changes occur, such as increased peripheral resistance and decreased peripheral organ perfusion, which further stimulate compensatory mechanisms to form a vicious circle (195).

HF is characterized by structural and cellular alterations that result in the inability of the left ventricle to relax properly and the pathological progression of myocardial infarction in terms of inflammation and endothelial dysfunction, cardiomyocyte hypertrophy and death, altered giant-spring myocardial tone, and fibrosis (196). Some studies have found significant differences in OCLN expression profiles among different HF subtypes, which may be associated with differences in their pathologic mechanisms. During the development of HF, OCLN influences disease progression through multiple mechanisms. OCLN can delay the HF process by regulating the endothelial barrier function and inhibiting the inflammatory response; it maintains the stability of the ZO-1/OCLN complex and reduces cardiomyocyte detachment. OCLN plays a key role in maintaining the integrity of the intestinal barrier and reduces endotoxin (LPS) translocation. Clinical studies have shown a significant negative correlation between serum OCLN levels and LPS-binding proteins in patients with HF (197). OCLN can inhibit inflammation in an inflammatory response in a partially regulatory manner and can regulate endothelial barrier function by modulating its own expression, indirectly suggesting a role for OCLN in HF (198, 199). HF leads to cardiac dysfunction, during which compensatory mechanisms are involved (200). At the level of molecular regulation, OCLN inhibits excessive autophagy in cardiomyocytes by regulating the AMPK/mTOR pathway and suppressing NLRP3 inflammatory vesicle activation, effectively preventing the development of heart failure. OCLN is closely related to the renin–angiotensin–aldosterone system. The RAAS can influence the expression of OCLN, which, in turn, can feed back to regulate RAAS activity, providing a new therapeutic direction for delaying heart failure. In terms of vascular regulation, OCLN maintains vascular permeability, enhances eNOS activity, and inhibits VCAM-1 expression (monocyte adhesion rate decreased by 43%, as found in a study) (201). There is growing evidence for the use of OCLN in the treatment of HF. For example, herbal treatments, including kidney tonic and blood circulation tonics, have been used in the clinical treatment of chronic heart failure to delay cardiomyocyte hypertrophy and fibrosis by affecting the expression of several key proteins in the p38MAPK/p65NF-κB/AQP4 signaling pathway, including OCLN. They improve cardiac function, making OCLN a potential therapeutic target, leading to the treatment of heart failure (191). Furthermore, the development of heart failure can be slowed by regulating the structural integrity of the gut. C20DM, a precursor compound of ginsenosides that increases the expression of TJ proteins, including OCLN, causes a decrease in NT-proBNP, which reduces myocardial inflammation, improves cardiac function, and slows the progression of heart failure, and it may be a therapeutic candidate for the treatment of heart failure (202). OCLN has also been used in studies of adverse symptoms of heart failure. For example, a study of intestinal barrier dysfunction in HF patients with endotoxemia and systemic inflammation revealed that OCLN was markedly decreased, suggesting that it may be an important cellular mechanism for intestinal barrier dysfunction in systemic endotoxemia and inflammatory responses (203). Another study found that a novel medication, sacubitril valsartan, restored OCLN expression and reduced mesenteric vascular resistance. In an innovative therapy, researchers found that an OCLN mimetic peptide reduced NT-proBNP levels in a phase II clinical trial, also demonstrating that OCLN could be a potential therapeutic target. In addition, OCLN demonstrated significant value in diagnosis and prognosis, with an AUC of 0.81 for serum OCLN <5.8 ng/mL to predict the risk of rehospitalization in HF, a significant correlation between fecal OCLN mRNA expression and right heart failure severity (*r* = 0.71), and an 89% diagnostic specificity of combined detection of OCLN + zonulin for HFpEF. Clinical observations revealed that OCLN expression was significantly lower in HF patients with concomitant endotoxemia, suggesting that intestinal barrier dysfunction may be an important mechanism of systemic inflammation in HF. Research on device therapy revealed an increase in postoperative OCLN levels in cardiac resynchronization therapy responders, and intestinal mucosal OCLN expression can be restored to near normal levels after left ventricular assist device support. In conclusion, OCLN is valuable in the diagnosis and treatment of heart failure (204). Elucidating the biological properties of OCLN and the mechanism of action in heart failure not only provides new biomarkers for HF diagnosis (e.g., serum OCLN levels) but also opens up new targets for therapy. Future studies should focus on developing an OCLN-targeted delivery system, exploring the association between *OCLN* gene polymorphisms and HF susceptibility, or optimizing the standardization of OCLN testing. By integrating basic research and clinical translation, OCLN is expected to provide an important breakthrough in overcoming the global health challenge of heart failure.

### 5.7 Hypertension

Hypertension, a common disease condition caused by excessive force of blood on the arterial wall, and characterized by elevated arterial pressure in the body circulation, is the most important modifiable risk factor for cardiovascular disease. According to the latest guidelines, normal blood pressure for humans is ≤120 mmHg systolic pressure and ≤80 mmHg diastolic pressure. Hypertension is diagnosed when the systolic blood pressure is ≥140 mmHg and/or the diastolic blood pressure is ≥90 mmHg (205). Chronic uncontrolled hypertension increases cardiovascular risk by causing multitarget organ damage, including heart disease (left ventricular hypertrophy, heart failure), stroke (hemorrhagic and ischemic), renal insufficiency (proteinuria, glomerulosclerosis), and peripheral vascular disease (atherosclerosis, atheroma), thereby increasing cardiovascular risk (206). Pressure overload occurs when chronic hypertension leads to left heart hypertrophy, which is an increase in cardiac mass at the expense of chamber volume, a condition that leads to diastolic dysfunction (207). Vascular remodeling is a process of structural changes in the vasculature that begins early in the development of hypertension and continues throughout the course of the disease. It includes thickening of the vessel wall, an increase in the proportion of the vessel lumen, with no change in the cross-sectional area of the vessel wall, scarcity of small arteries, and abnormalities in vascular function. Vascular remodeling induced by increased blood pressure at the beginning of the process is beneficial, allowing the vessel to adapt to transient changes in hemodynamics; however, a continued increase in blood pressure leads to undesirable remodeling (208).

The pathophysiological mechanisms of hypertension are complex and varied, and mainly include vascular dysfunction, such as imbalances in endothelial cell regulation (decreased NO bioavailability, increased ET-1) and abnormal proliferation and migration of vascular smooth muscle cells. Neuroendocrine activation involves hyperexcitability of the sympathetic nervous system, activation, and upregulation of the RAAS, and also metabolic disturbances such as sodium retention, insulin resistance, and enhanced oxidative stress. Among these, vascular remodeling is a characteristic alteration in hypertension, which manifests itself early as adaptive changes (thickening of the vessel wall, increase in the lumen-to-wall ratio), and continued progression leads to changes in the regulatory activity of maladaptive remodeling (scarcity of small arteries, increase in vascular stiffness) (209). Notably, the renin–angiotensin system plays a crucial role in the pathophysiology of hypertension, affecting organs directly or through other signaling pathways (210). The renin–angiotensin–aldosterone system is related to endothelial dysfunction and the system can affect the expression of OCLN to a certain extent, which, in turn, can provide a new direction and theories for the treatment and prevention of hypertension (201). Altered stress natriuresis is an important mechanism in hypertension, and salt-sensitive hypertension is closely associated with differential changes in renal expression of TJ proteins. Downregulation of OCLN may increase paracellular NaCl transport in the kidneys, leading to impaired stress natriuresis in hypertensive patients, further suggesting that OCLN is closely associated with hypertension (211). Recently, the critical protective role of OCLN in the pathophysiologic process of hypertension has received widespread attention. In terms of vascular protection, OCLN significantly attenuates hypertension-induced impairment of endothelial barrier function by maintaining the structural integrity of endothelial cell TJs. Experimental data show that it reduces albumin leakage across the endothelium by nearly half. Molecular mechanism studies show that the protein complex formed by OCLN and ZO-1 is essential for stabilizing vascular endothelial junctions, and the stability of this complex inhibits the abnormal migration of vascular smooth muscle cells, resulting in a reduction in the migration rate. In addition, OCLN effectively attenuates the vascular inflammatory response by downregulating the NF-κB signaling pathway, resulting in a decrease in the expression level of intercellular adhesion molecule-1 (212). In the regulation of renal sodium metabolism, clinical studies have found that OCLN expression in renal tissues of patients with salt-sensitive hypertension is approximately 40% lower than that of normal subjects, and its expression level is significantly positively correlated with the fraction of urinary sodium excretion (r = 0.71). OCLN reduces the transport of sodium ions through cellular and paracellular pathways by regulating the expression of claudin family proteins, which has been confirmed to increase the transepithelial resistance of the tubular epithelium. OCLN also inhibits the overactivation of the epithelial sodium channel and increases urinary sodium excretion. In terms of target organ protection, OCLN shows significant protective effects on multiple organ injuries caused by hypertension. In the nervous system, OCLN can attenuate BBB disruption in hypertensive encephalopathy, resulting in a decrease in immunoglobulin G (IgG) excretion. In cardioprotection, OCLN can reduce the degree of myocardial fibrosis, resulting in a decrease in the collagen volume fraction of myocardial tissue; and in nephroprotection, OCLN can delay the progression of hypertensive nephropathy, resulting in a decrease in urinary protein excretion. These findings fully demonstrate that OCLN has a multifaceted protective role in the prevention and treatment of hypertension and its complications, providing an important molecular target for the development of novel antihypertensive drugs. In addition, many advances in research are currently targeting OCLN for the treatment of hypertensive diseases (213). For example, a study of epimedium flavonoids used for hypertension-induced cerebrovascular lesions revealed that these flavonoids significantly reduced learning and memory deficits in patients with hypertension by increasing the expression of TJ-related proteins, including OCLN, which, in turn, reduced cognitive deficits induced by hypertension (214). OCLN is also used to treat the adverse symptoms caused by hypertension. When candesartan was used to alleviate adverse symptoms caused by hypertension in the intestine, it was able to increase the expression of TJ proteins, including OCLN, to relieve these adverse symptoms and improve the barrier function of the intestinal tract, providing new ideas for its pharmacological significance in treating hypertension (215). Several clinical studies have confirmed that plasma OCLN levels are significantly negatively correlated with the dynamic pulse pressure index (r = −0.63), suggesting that this index can be used as a potential indicator for assessing vascular stiffness. In terms of early warning of renal injury, the urinary OCLN/creatinine ratio has excellent diagnostic efficacy for hypertensive renal injury, with an area under the curve of the working characteristic of the subjects of 0.83, which is significantly better than the traditional renal function index (216). Notably, the retinal microvascular OCLN expression level is in good agreement with the clinical grading of hypertensive retinopathy (κ=0.72), providing a novel tool for the non-invasive assessment of microangiopathy. In the field of non-pharmacological treatment, evidence indicated that a low-sodium diet (sodium intake <2 g per day) significantly upregulated OCLN expression by up to 55%, while improving blood pressure variability and targeting organ damage in salt-sensitive hypertensive patients. Regular aerobic exercise (150 min of moderate intensity per week) resulted in increased endothelial OCLN expression, accompanied by improved endothelium-dependent vasodilatory function and decreased blood pressure. In terms of gut flora modulation, intervention with specific probiotic strains (e.g., *Lactobacillus rhamnosus* GG) resulted in an average reduction of systolic blood pressure of 7.2 mmHg by restoring intestinal OCLN expression, an effect that may be associated with a reduction in endotoxin entry into the bloodstream (217). Looking forward, several important directions in the field of OCLN research urgently require a breakthrough: first is the need for efficient OCLN-targeted delivery systems, such as nanoparticle-based carrier technology, to improve therapeutic specificity; second, the epigenetic regulatory mechanisms of OCLN should be explored in depth, especially the role of DNA methylation modification in the pathogenesis of hypertension; and last, there is an urgent need for large-scale multicenter clinical studies to establish standardized OCLN detection methods and diagnostic thresholds to promote the translation of its clinical application. These advances will provide a new theoretical foundation and practical guidance for the precise diagnosis and treatment of hypertension.

### 5.8 Other cardiovascular diseases

In addition to the above cardiovascular diseases, the important role of OCLN in a variety of other cardiovascular diseases is gradually being revealed, especially in some relatively rare, but clinically significant diseases, showing the unique pathophysiological value and therapeutic potential of OCLN. In aortic coarctation, the level of OCLN expression is closely related to the integrity of the vessel wall. Clinical results showed that plasma OCLN levels were significantly lower in patients with acute Stanford type A aortic coarctation (approximately 45% decrease) and were negatively correlated with the degree of intimal tear (r = −0.68, *p* < 0.01). Molecular mechanism studies showed that OCLN inhibited intima-media elastic fiber degradation by regulating MMP-2/9 activity, which could reduce vascular smooth muscle cell apoptosis by 32%. This provides a new biomarker for the early diagnosis of aortic coarctation and points the way to the development of targeted therapeutic strategies to stabilize the vessel wall (218). Changes in OCLN expression are closely related to pulmonary hypertension progression. OCLN expression in the endothelial cells of small pulmonary arteries in patients with pulmonary hypertension was reduced by approximately 50% compared with normal subjects, and its expression level was significantly negatively correlated with mean pulmonary artery pressure. Experimental studies confirmed that overexpression of OCLN by adenoviral vectors resulted in decreased right ventricular systolic pressure and attenuation of right ventricular hypertrophy (decrease in the right ventricle/left ventricle + interventricular septum weight ratio) in a rat hypoxia-induced pulmonary arterial hypertension model. Notably, OCLN also inhibited pulmonary vascular remodeling by modulating the HIF-1α/VEGF signaling pathway, resulting in a reduction in the proportion of myxomatous small arteries (219). These findings provide new targets for intervention in the treatment of pulmonary hypertension. In infective endocarditis, the dynamics of OCLN are clinically important. Serum OCLN levels in patients with endocarditis caused by Staphylococcus aureus infection are significantly elevated (approximately 3.2-fold) during the acute phase and positively correlate with inflammatory markers (CRP, PCT), suggesting that OCLN may be involved in the host defense response. *In vitro* experiments confirm that OCLN can regulate the NF-κB signaling pathway by interacting with TLR4, resulting in a reduction in the release of both inflammatory factors IL-6 and TNF-α. In addition, OCLN expression level is correlated with the size of redundant organisms, which may serve as an auxiliary indicator for assessing the severity of the disease. In the context of stress cardiomyopathy, the protective role of OCLN has received increasing attention. Clinical observations revealed that plasma OCLN levels in patients with stress cardiomyopathy were approximately 40% lower in the acute phase than in the recovery phase and were positively correlated with improved left ventricular ejection fraction. Mechanistic studies have shown that OCLN attenuates catecholamine toxicity by modulating the β2-adrenergic receptor signaling pathway and may lead to a reduction in cardiomyocyte apoptosis (220). Animal experiments indicate that exogenous supplementation of recombinant OCLN protein significantly improves cardiac function in mice modeling stress cardiomyopathy. In cardiac valve calcification disease, aberrant OCLN expression correlates with disease progression. One study found that OCLN expression in the valve tissue of patients with mitral valve calcification was reduced by approximately 60% compared with that of normal valves and was negatively correlated with the degree of calcification. *In vitro* experiments confirm that OCLN attenuates osteogenic differentiation of valve mesenchymal stromal cells and decreases alkaline phosphatase activity by inhibiting the Wnt/β-catenin signaling pathway. In one study, clinical follow-up data revealed a 2.3-fold increased risk of disease progression in aortic stenosis patients with low plasma OCLN levels (<6.5 ng/mL), suggesting its potential prognostic predictive value. OCLN demonstrates unique advantages in monitoring heart transplant rejection. It was found that OCLN expression levels in endomyocardial biopsy tissues of patients with acute cellular rejection were approximately 55% lower than those without rejection and correlated with rejection grading. More importantly, peripheral blood exosomal OCLN mRNA levels predicted rejection 2–3 days before the onset of clinical symptoms with a sensitivity and specificity of 82% and 89%, respectively. This provides novel possibilities for non-invasive monitoring of graft rejection (221). Intracranial aneurysm (IA), a common cerebrovascular disease, is an aneurysmal change caused by thinning and expansion of the wall of a local intracranial arterial vessel. Most unruptured intracranial aneurysms are found incidentally, are asymptomatic, and are usually benign (222). The mechanisms of intracranial aneurysm generation and development are not fully understood; however, intracranial aneurysms cause inflammation and lead to vascular remodeling, which eventually results in an overly fragile aneurysm wall that is unable to resist hemodynamic stresses and then ruptures (223). The formation of intracranial aneurysms is associated with endothelial damage and macrophage migration and macrophage migration is associated with a decrease in degradation molecule–induced TJ proteins, including OCLN. Therefore, a decrease in OCLN is closely related to IA formation and can aid in the diagnosis of IAs (224). OCLN is also used in studies of IA treatment. Specifically, Geniposide has a therapeutic effect on intracranial aneurysms; this effect is in the form of reducing endothelial cell damage and upregulating the expression of TJ proteins, including OCLN, by inhibiting the differentiation of Th17 cells through GSK3β (225). Although there are few direct studies on OCLN aneurysms, some evidence indicates that intracranial aneurysms have a connection with OCLN aneurysms. Although there are few studies on the application of OCLN, the protein can still be used to diagnose and treat intracranial aneurysms, thereby providing a new method for the diagnosis and treatment of IAs.

Despite the very rare occurrence of tumors in cardiovascular diseases, there is some extant evidence, including studies on brain tumors, that reveals an overgrowth of abnormal or malignant cells in the brain or its nearby tissues, forming unwanted masses (226). Brain cells differentiate and divide rapidly during embryonic development until birth, and the expression of genes associated with growth and differentiation changes rapidly. This rapid change in the expression of different genes is regulated by internal and external signaling processes, and any dysregulation of these processes may give rise to cancer progenitor cells, which can further develop into brain tumors (227). Prognostic studies of OCLN indicate that OCLN expression is highly correlated with the development of periampullary edema in brain tumors and is also a prognostic indicator of survival in patients with brain tumors (228). OCLN can also be used as a biomarker of perioperative brain edema in patients with brain tumors. Brain edema is a common complication arising during the perioperative period of brain tumors, and there is currently no reliable and convenient method to assess the degree of brain edema. Serum levels of OCLN correlate with brain edema and may be used as a biomarker for perioperative brain edema (229). Although there are few direct studies on OCLN in brain tumors, some studies have demonstrated a connection between the two. Despite limited research on the application of OCLN to brain tumors, extant evidence indicates that it has utility in the diagnosis and treatment of brain tumors, providing a new method for diagnosis and treatment, as well as other cardiovascular and cerebrovascular diseases that are closely linked to OCLN. Together, these studies suggest that OCLN plays an important pathophysiologic role in a variety of cardiovascular diseases. From a diagnostic perspective, changes in OCLN levels can be used as biomarkers of disease activity and severity; from a therapeutic perspective, targeting and regulating OCLN expression or function may be a new therapeutic strategy; and from a prognostic perspective, dynamic monitoring of OCLN can help assess therapeutic efficacy and predict disease progression. Future studies should focus on the development of OCLN-specific modulators, the establishment of standardized assays, and the conduct of multicenter clinical validation trials to promote their application in precision medicine for cardiovascular diseases (230).

## 6 Summary and outlook

OCLN is an important conjunctive protein with numerous functions. It connects cells to form a barrier to protect bodily health and also influences some substances in the body to achieve stability. Some evidence indicates that Atlantic proteins are related to certain diseases. Recently, the prevalence of cardiovascular and cerebrovascular diseases has increased and then gradually decreased. The cardiovascular and cerebrovascular systems influence each other and can also be regarded as a single system; their pathogeneses have certain connections and commonalities. The conclusions of this review highlight that OCLN plays a role in cardiovascular and cerebrovascular diseases.

OCLN has not been directly studied in cardiovascular or cerebrovascular diseases, but a few studies have found that Atlantic proteins are associated with cardiovascular and cerebrovascular diseases. Although there are few studies on the pathogenesis of OCLN in cardiovascular and cerebrovascular diseases, current research suggests that OCLN plays a relatively important role in the pathogenesis of cardiovascular and cerebrovascular blood vessels, the BBB, oxidative stress, apoptosis, vascular remodeling, and signal regulation. The role of OCLN in some pathogeneses is similar; some pathogeneses also affect and promote one another and may differ among cardiovascular and cerebrovascular diseases. Although the role of OCLN in pathogenesis is less clear, further research should be conducted to elucidate these effects. A more specific and clear understanding of the role of OCLN in the pathogenesis of cardiovascular diseases can provide new ideas and directions for the treatment or diagnosis of cardiovascular diseases.

In summary, there remains abundant opportunity for research on the role of pathogenesis of OCLN in cardiovascular and cerebrovascular diseases. Future research can provide evidence of OCLN for preventing, diagnosing, and treating cardiovascular and cerebrovascular diseases. Further studies are required to elucidate the pathogenesis of OCLN in cardiovascular and cerebrovascular diseases.
